# Genome Deletions and Rewiring of the Transcriptome Underlying High Antimonite Resistance in *Achromobacter* sp. SMAs-55

**DOI:** 10.3390/ijms26010107

**Published:** 2024-12-26

**Authors:** Yanshuang Yu, Martin Herzberg, Aurora M. Pat-Espadas, Pablo Vinuesa, Renwei Feng, Barry Rosen, Seigo Amachi, Xianbo Jia, Christopher Rensing, Shungui Zhou

**Affiliations:** 1 College of Resources and Environment, Fujian Agriculture and Forestry University, Fuzhou 350002, China; yuyanshuang2018@163.com (Y.Y.); frwzym@aliyun.com (R.F.); sgzhou@fafu.edu.cn (S.Z.); 2 Institute of Resources, Environment and Soil Fertilizer, Fujian Academy of Agricultural Sciences, Fuzhou 350002, China; xbj2011@163.com; 3 Department of Solar Materials Biotechnology (SOMA), Helmholtz Centre for Environmental Research—UFZ, 04318 Leipzig, Germany; martin.herzberg@ufz.de; 4 CONAHCYT-Institute of Geology, Estación Regional del Noroeste, Universidad Nacional Autónoma de México, Luis Donaldo Colosio s/n, Hermosillo 83250, Sonora, Mexico; aurorampatespadas@gmail.com; 5 Centro de Ciencias Genómicas, Universidad Nacional Autónoma de México, Cuernavaca 62210, Morelos, Mexico; vinuesa@ccg.unam.mx; 6 Department of Cellular Biology and Pharmacology, Herbert Wertheim College of Medicine, Florida International University, Miami, FL 33199, USA; brosen@fiu.edu; 7 Graduate School of Horticulture, Chiba University, Matsudo 271-8510, Japan; amachi@faculty.chiba-u.jp

**Keywords:** *Achromobacter*, metalloids, antimony, arsenic, genome plasticity, transcriptome

## Abstract

Microbes have been shown to adapt to stressful or even lethal conditions through displaying genome plasticity. However, how bacteria utilize the ability of genomic plasticity to deal with high antimony (Sb) stress has remained unclear. In this study, the spontaneous mutant strain SMAs-55 of *Achromobacter* sp. As-55 was obtained under antimonite (Sb(III)) stress. SMAs-55 displayed significantly increased Sb(III) resistance, but it lost the ability to oxidize arsenite (As(III)) by deleting an entire gene island containing genes encoding functions involved in As(III) oxidation, arsenic (As)/Sb resistance and phosphate transport. This study suggests that genetic plasticity has played an important role in As-55 adaption to Sb(III) stress. Transcriptomic analysis found that genes encoding functions involved in capsule polysaccharide synthesis, as well as functions correlated to stress adaptation, ATP production, and metabolism were more strongly expressed in SMAs-55. In addition, a lower intracellular Sb(III) accumulation in SMAs-55 was observed. These findings indicate that reduced uptake through increased capsule biosynthesis was an effective way for SMAs-55 to adapt to an environment displaying high levels of Sb. This study helps us to better understand the evolutionary processes enabling survival of microbes and microbial community in contaminated environments.

## 1. Introduction

Antimony (Sb) and Arsenic (As) are highly toxic metalloids that often coexist in nature [1,2]. Environmental contamination by As and Sb is a global issue of great concern. As and Sb have been shown to enter the human body through drinking water and the food chain [3,4]. Both As and Sb are able to inhibit the activity of hundreds of enzymes, activate carcinogens, and damage the nervous and respiratory systems, thereby endangering human health. Exposure to As and Sb causes neurological diseases, immune diseases, cardiovascular diseases, skin diseases, and even cancers [5,6]. As and Sb also have toxic effects on many plants, not only affecting plant growth and development by affecting plant photosynthesis and anti-oxidative damage [7,8,9,10], but also affecting soil health through toxic effects on soil microorganisms, thereby influencing the yield and quality of agricultural products [11,12].

Although arsenic and antimony pollution poses an increasing threat to human and environmental health, some microorganisms have been shown to survive in environments with high concentrations of arsenic and antimony [1,13,14,15,16], and even oxidize arsenic and antimony as energy substrates. Microorganisms in mining areas have evolved a number of adaptive mechanisms, such as As/Sb oxidation [17,18], reduction [19], and methylation [20,21] in high As- and Sb-polluted environments to cope with such extreme conditions [16], thereby playing a vital role in the migration and transformation of As and Sb [22]. Microbe-mediated As-transformations have been well studied: for example, arsenate reduction by ArsC or ArrAB, arsenite oxidation by AioAB [17] and ArxAB [23,24], organic arsenical oxidation by ArsH [25]/ArsU [26]/ArsZ [27]/ArsV [18], and arsenite methylation by ArsM [28]. While the information on Sb transformation is quite sparse, with only reporting reduction by ArrAB [19], oxidation by AioAB [17] and efflux by ACR3/ArsB/ArsK [29,30] which also present corresponding resistance to As and transformation of As. Distinct resistance mechanisms of Sb still need more exploration.

Recently, more and more arsenite oxidizers have been isolated and identified because microbe-mediated arsenite oxidation has been demonstrated to be an eco-friendly and cost-effective method for bioremediation of arsenic contamination [20,22,31]. So far, As(III) oxidation has only been applied and studied with of a single As contamination. In natural environments, the contamination is complex, and the contamination of both As and Sb is common [1], especially in As or Sb mining areas. Therefore, microorganisms that are able to oxidize As in the presence of Sb are required. In our previous enrichment experiments, we isolated multiple As and Sb resistant bacteria. We found that As resistance is not always linked with Sb resistance, with strains exhibiting very high As resistance, but very low Sb resistance. Similarly, the arsenite oxidizer also showed very low resistance to Sb. Unlike the other microbes which have similar resistance mechanisms to As and Sb, arsenite oxidizers carrying classical *ars* operon which confer As/Sb resistance still exhibited very low resistance to Sb. The correlation between As(III) oxidation, Sb(III) resistance and underling mechanisms remain unclear. Therefore, deciphering microbial mechanisms which can adapt to the exposure of high concentrations of Sb is of great practical significance to reveal how As resistant microbes with low Sb resistance persist and remain active in this complex environment.

In this study, a strain As-55 of *Achromobacter* exhibiting high arsenite resistance and strong arsenite oxidation ability, but low resistance to antimonite was isolated from a corn farm near the Xikuangshan antimony mine, contaminated with arsenic and antimony. Interestingly, a mutant of As-55 with an increased antimonite resistance was obtained under antimonite exposure. Characterization and genomic analysis of wild-type strain As-55 and mutant SMAs-55 were performed to decipher changes in arsenite resistance, oxidation, and a potentially new antimonite resistance mechanism at the molecular level.

## 2. Results

### 2.1. Identification and Characterization of Strain As-55

A total of 115 strains were isolated by As and Sb enrichment. One isolate showing As(III) oxidation ability was obtained by screening all of the isolates using the silver nitrate method and was, therefore, chosen for further study. Compared to the other four As(III) oxidizers, previously isolated and characterized [32,33] and also belonging to the genus *Achromobacter*, strain As-55 displayed higher resistance to As(III), and was able to grow at 35 mM As(III) on R2A agar medium, while the other isolates were only able to grow on R2A medium containing 20 mM As(III) (Supplementary Figure S1A). Correspondingly, the As(III) oxidation capability of As-55 was much higher than those of the other four *Achromobacter* strains (Supplementary Figure S1B). In addition, the As(III) resistance of As-55 was inducible (Supplementary Figure S1C,D). Moreover, As-55 displayed chemotaxis towards As (Supplementary Figure S1K) and also grew well under the exposure of low concentration of arsenic/copper/cadmium (Supplementary Figure S1E,G,H). Upon exposure to high concentrations of As(III), As-55 grew significantly and performed As(III) oxidation (Supplementary Figure S1I,J). Although As-55 exhibited a low initial resistance to Sb(III), As-55 was able to adapt to higher Sb(III) concentrations (Supplementary Figure S1F), indicating that As-55 represents a good model for studying processes of adaptation within the framework of a laboratory evolution approach, leading to higher antimony resistance.

### 2.2. Phenotypic Changes in the Spontaneous Mutant Strain SMAs-55

**Figure 1:**
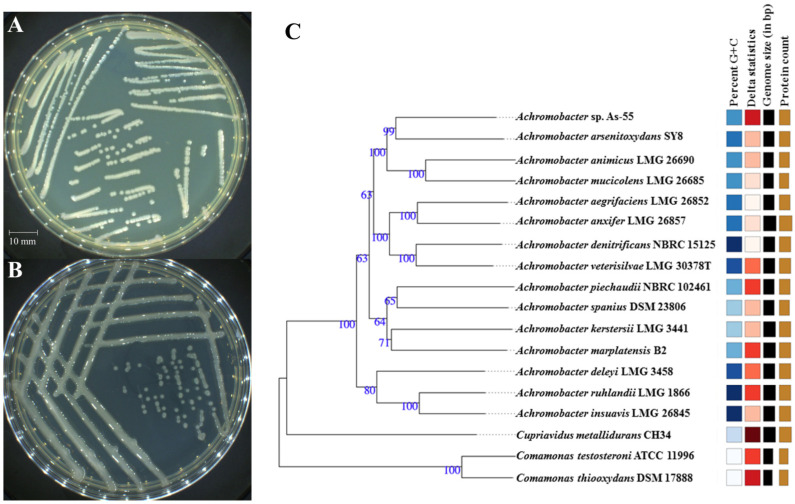
Morphology of wild-type As-55 (**A**) and spontaneous mutant SMAs-55 (**B**) on LB agar medium displaying a more mucoid phenotype of SMAs-55, and a molecular phylogenetic tree based on the MASH algorithm and 16S rRNA gene sequences, highlighting the position of *Achromobacter* sp. As-55 relative to other type and non-type strains of the genus *Achromobacters* and *Comamonas* (**C**). The evolutionary history was inferred by MASH and 16S rDNA-based tree with the Type (Strain) Genome Server (TYGS) uses the Genome BLAST Distance Phylogeny (GBDP) provided by Leibniz Institute DSMZ (https://tygs.dsmz.de/, accessed on 24 June 2024), for a whole genome-based taxonomic analysis. Color code: Percent G+C—the darker the shade, the higher the G+C content. Delta statistics—provide guidance regarding the suitability of specific query genome sequences and the reliability of the phylogenetic outcome. Genome size and protein counts—the bar length reflects the relative size between the used species and strains.

To unravel the mechanisms underlying the adaptation of As-55 to Sb(III), We exposed As-55 to a high concentration of Sb(III), and after one week of exposure a spontaneous mutant of As-55, referred as SMAs-55, was obtained. The morphology of SMAs-55 changed significantly compared to that of As-55 on LB agar medium, exhibiting a mucoid colony phenotype (Figure 1A,B), suggesting an increased secretion of extracellular polymers in SMAs-55 (Supplementary Figure S2). The 16S rRNA gene sequence of SMAs-55 was shown to be 100% identical to As-55, excluding the possibility of contamination. Based on the sequence alignment results of the entire genome, strain As-55 and *Achromobacter arsenitoxydans* SY8 aggregate to form a small branch, as shown in Figure 1C. Therefore, the ANI values of strains As-55 and SY8 were calculated using the online tool on Eztaxon website, which was 88.42%, less than the defined value for distinct species (95%) [34]. It was concluded that strain As-55 may be a representative of a new *Achromobacter* species.

### 2.3. Physiological Characterization of As-55 and Spontaneous Mutant SMAs-55

As-55 is an aerobic, mobile, rod-shaped Gram-negative bacterium. After incubation on LB agar at 28 °C for 48 h, the colonies displayed smooth, translucent, and non-pigmented margins (Figure 1A, Supplementary Figure S3). For the mutant SMAs-55 under the same culture conditions, the colonies showed more mucoid, suggesting EPS production (Figure 1B). BioMérieux enzyme activity measurements for As-55 and SMAs-55 are shown in Supplementary Table S1. The wild-type strain As-55 exhibited alanine phenylalanine proline aromatase (APPA), L-proline aromatase (ProA), tyrosine aromatase (TyrA), lactate alkali production (lLATk), succinate alkali production (SUCT), ELLMAN (ELLM), citrate (sodium) (CIT), and L-malate assimilation (MLTa) enzyme activity. However, the spontaneous mutant SMAs-55 lost citrate (sodium) (CIT) and L-malate assimilation (MLTa) enzyme activity, and increased glutamyl aromatase (AGLTp) activity.

### 2.4. Changes in As(III)/Sb(III) Resistance and the Ability to Oxidize As(III) Occurred When Comparing As-55 and the Spontaneous Mutant SMAs-55

**Figure 2:**
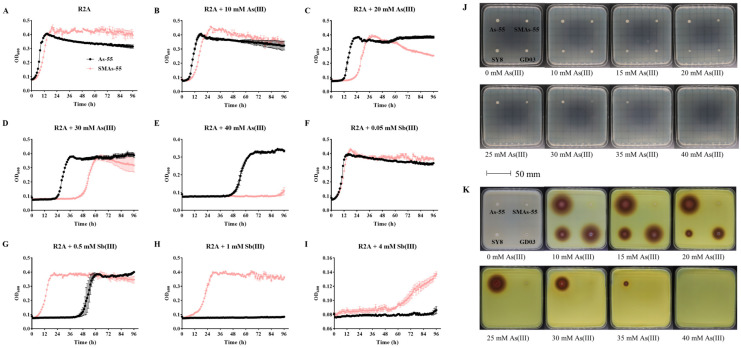
Phenotypic As(III) and Sb(III) resistance and ability to oxidize As(III) under differing As(III) concentrations. Growth curve of wild-type (As-55) (black filled circles) and spontaneous mutant (SMAs-55) (colored diamond) in R2A medium with As(III) (**B**–**E**), Sb(III) (**F**–**I**) and without metals (**A**). As-55, SMAs-55, *Achromobacter arsenitoxydans* SY8 and *Achromobacter xylooxidans* GD03 (positive controls with As(III) oxidation ability) cultured on R2A medium containing different concentrations of As(III) for 2 d (**J**). Plates flooded with 0.1 M AgNO_3_ and reaction in dark condition for 2 min (**K**). Brown-dark precipitates around individual colonies indicated a positive As(III) oxidation reaction.

Compared to the wild-type strain As-55, the spontaneous mutant strain SMAs-55 displayed a significant increase in Sb(III) resistance (Figure 2). After exposure to 0.5 mM Sb(III), SMAs-55 grew rapidly (Figure 2G), while the wild-type strain As-55 required a longer amount of time (48 h) to adapt to this concentration of Sb(III). Under high concentrations (1 mM) of Sb(III), As-55 was unable to grow in the time frame of 96 h, whereas SMAs-55 was able to maintain a biomass equivalent to that obtained in Sb(III)-free medium (Figure 2H), and was even able to grow at concentrations up to 4 mM Sb(III) (Figure 2I). The As(III) and Sb(III) resistance of SMAs-55 displayed different trends. Under low concentrations of As(III) (10, 20 and 30 mM), the growth of SMAs-55 was weaker than that of the wild-type strain As-55, and its lag phase (the time when the OD_600_ value reached 0.2) was longer than that of As-55 (Figure 2B–D). In the case of high concentrations of As(III) (40 mM) addition, SMAs-55 did not grow at all whereas As-55 began to grow and proliferate after 48 h of adaptation (Figure 2E).

The silver nitrate chromogenic results for the spontaneous mutant strain SMAs-55 are shown in Figure 2K. SMAs-55 displayed no oxidation of As(III) on a plate containing silver nitrate, indicating a complete loss of As(III) oxidation ability. However, the parental strain As-55 showed strong As(III) oxidation on a plate containing 10, 15, and 20 mM As (III), and its oxidation circle was larger than other members of the same genus of the control strains *Achromobacter arsenitoxydans* SY8 [33] and *Achromobacter xylosoxidans* GD03 [32] cultured simultaneously under the same conditions (Figure 2K). As the concentration of As(III) further increased to 25, 30, and 35 mM, the control strains SY8 and GD03 were unable to grow, whereas As-55 still showed strong growth and As(III) oxidation ability (Figure 2J,K). Although SMAs-55 grew, its growth was decreased with increasing As(III) concentrations than that of the wild-type strain As-55.

### 2.5. Genomic Analysis Illustrated Significant Changes in Spontaneous Mutant SMAs-55 When Compared to As-55

**Figure 3:**
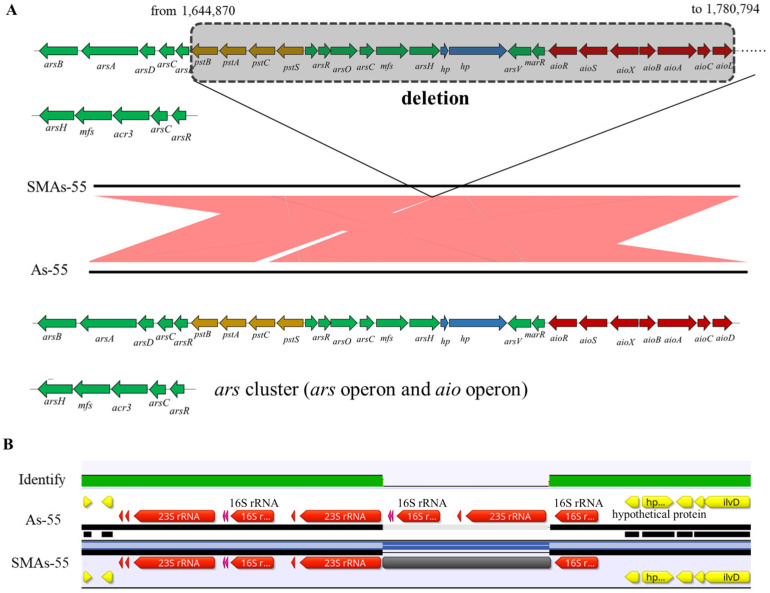
Genomic comparison of As-55 and SMAs-55. The fragment containing the deletion of a gene island containing *ars*/*aio*/*pst* operon with shadow (**A**) and a set of ribosomal RNA (16, 5, 23S RNA) occurring in SMAs-55 (**B**).

To better analyze potential genetic changes underlying the loss of the ability to oxidize As(III) and the increased resistance to Sb(III), the complete genomes of As-55 and SMAs-55 were sequenced. The results of the comparative genome analysis between the spontaneous mutant strain SMAs-55 and the wild-type strain As-55 are shown in Supplementary Table S2. There are four missing fragments on the genome of SMAs-55, with the largest deleted fragment (135,925 bp) being annotated as an entire gene island, containing an operon *aioXRSABCD* encoding functions necessary for As(III) oxidation, an arsenic resistance operon *arsROC*-*mfs*-*arsH*, and an operon *pstSCAB* encoding a high affinity phosphate transport system (Figure 3A), which explains the loss of As(III) oxidation ability of the spontaneous mutant strain at the genetic level. The second-largest deleted fragment had a length of 5994 bp and included a ribosomal RNA operon *rrn* (tRNA Ala, tRNA Ile, 16S, 5S, and 23S rRNA) (Figure 3B). The deletion of the other two shorter sequences occurred on the gene encoding the retention module containing protein (HLG70_12305), which belongs to the general function prediction only category in COG classification. The mechanism of its impact on the adaptation of SMAs-55 to a high Sb(III) environment requires further experimental exploration.

### 2.6. Different Stress Response of As-55 and SMAs-55 Under Exposure to As(III) or Sb(III)

To elucidate the molecular mechanisms underlying key epigenetic changes for surviving under high Sb(III) stress at the transcriptomic level, the transcriptome sequencing of As-55 and SMAs-55 was performed with and without Sb(III) or As(III) addition. The Pearson correlation coefficients within each sample group were all >0.9 (Supplementary Figure S4A), showing good biological repeatability and parallelism of the samples. Moreover, the correlation coefficient between the SMAs-55 and As-55 treatment groups was relatively small, indicating a significant difference in the expression levels between the two strains. In the PCA results, the distance between the treatment and control groups of the two bacteria was relatively large, indicating that there was a certain difference in gene expression between the two strains under the same conditions. The distance between the WSb0_5 group in As-55 and the other treatment groups was relatively far (Supplementary Figure S4B), possibly because As-55 itself displayed a lower resistance to Sb(III), resulting in a more extensive gene response under 0.5 mM Sb (III) stress compared to other treatments. However, the distance of the treatment of SSb_0.5 in SMAs-55 and other treatment groups was relatively close, which might have been due to a higher Sb(III) resistance of SMAs-55, and under the same conditions, its transcriptome response was more similar to that of other treatment groups in accordance with the MIC results of As-55 and SMAs-55.

General gene expression was evaluated using differentially expressed genes (DEGs). As the concentration of As(III) and Sb(III) increased, the number of DEGs also increased (Supplementary Figure S5) in a concentration-dependent manner. Notably, the number of DEGs under low Sb exposure in SMAs-55 was less than that in As-55.

For the wild-type strain As-55, As(III) oxidation was an important detoxification strategy under As(III) exposure (Supplementary Figure S6). The As(III) oxidase coding gene *aioAB* and the downstream structural gene of oxidation operon (*aioCD*) were upregulated by the highest fold in the As(III) treatment group, followed by the operon encoding the DUF4148 domain protein and the operon *arsRCDAB* (Supplementary Figure S6B,D). Whereas the primary detoxification mechanism of As-55 under Sb(III) stress was efflux. Although the As(III) oxidation operon was also induced, the most induced expression was mainly the efflux operon *arsRCDAB* (Supplementary Figure S6), following the operon encoding the DUF4148 domain protein and *arsV*.

For the spontaneous mutant SMAs-55, the resistance mechanism under As(III) and Sb(III) stress is predicted to be ATP-dependent efflux. Genes present on the *arsRCDAB* operon were the most upregulated under As(III) and Sb(III) stress, followed by the operon encoding the DUF4148 domain protein (Supplementary Figure S7). The ATPase ArsA had been shown to form an ATP-dependent efflux pump together with ArsB [35] indicating both Sb(III) and As(III) resistance is mostly due to efflux since there also is a subsequent loss of the operon encoding As(III) oxidase (Figure 3A). In addition, under high concentrations of As(III) (2 mM) stress, the top10 upregulated genes in treatment group SAs2vsSCK were a gene cluster encoding ABC transport substrate binding protein, phosphopyruvate decarboxylase, aldehyde dehydrogenase, aldehyde dehydrogenase family protein, and NAD(P)-dependent oxidoreductase (HLG70_04235-04255). Under high concentrations of Sb(III) (0.5 mM) stress, the top10 upregulated genes in the SSb0_5vsSCK group are involved in an operon encoding tartrate dehydrogenase, triplet tricarboxylic acid transporter substrate binding protein, and phosphatase PAP2 family protein (HLG70_27720-27730) (Supplementary Figure S7).

### 2.7. Transcriptomic Comparison Analysis Between As-55 and SMAs-55

**Figure 4:**
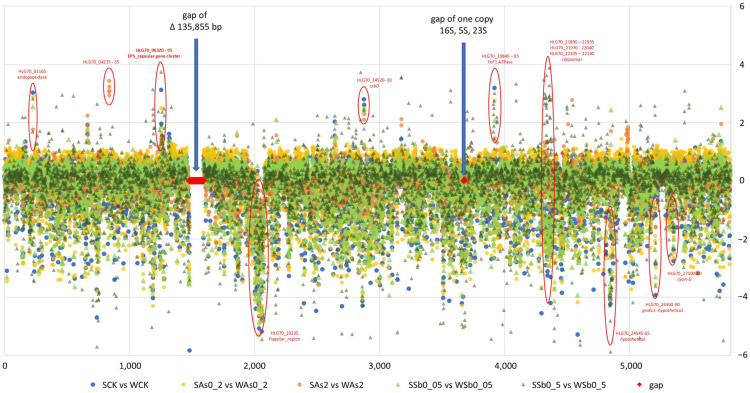
Comparative analysis of the SMAs-55 and As-55 transcriptome. SMAs-55 under treatment with 0.2 mM (yellow) and 2 mM (orange) As(III) or 0.05 mM (light green) and 0.5 mM (dark green) Sb(III) compared to As-55 under the same treatments. The ordinate is the log_2_ (fold change) value based on FPKM.

To display the gene expression changes in the spontaneous mutant SMAs-55 compared to the wild-type strain As-55, a transcriptomic comparison was performed between spontaneous mutant SMAs-55 and the wild-type strain As-55 under the same conditions. The overall changes in SMAs-55 relative to those in As-55 are shown in Figure 4. The gene clusters with significant changes in gene expression levels are circled in red, and specific differences are shown in Supplementary Table S3.

In all five comparison groups (SCKvsWCK, SAs0_2vsWAs0_2, SAs2vsWAs2, SSb0_05vsWSb0_05, and SSb0_5vsWSb0_5), an increased expression of gene (HLG70_01165) encoding endopeptidase, gene clusters encoding functions involved in cell wall/membrane/envelope biosynthesis and capsule biosynthesis (HLG70_06320~06390), and gene clusters encoding CsbD family proteins, DUF1328 domain proteins and BON domain proteins (HLG70_14520~14530) were observed.

While reduced expression was observed in gene clusters encoding flagellar synthesis and cell chemotaxis (HLG70_10070~10420); gene clusters encoding hypothetical proteins, metal phosphatases, and antitoxin VbhA family proteins (HLG70_2454545~24570); gene clusters encoding hypothetical proteins, autotransporter outer membrane β barrel domain proteins (HLG70_26380~26390) and genes encoding cytochrome o panthenol oxidase subunits IV, III, I, panthenol oxidase subunit II, and MFS transporters (HLG70_27100~27120) (Figure 4).

The increased expression of the gene cluster encoding aldehyde dehydrogenase (HLG70_04235-04255) was only observed in the high concentration As(III) comparative group (SAs2vsWAs2), and there was no significant change in other comparative groups. The increased expression of gene clusters encoding the F_0_F_1_ type ATP synthase subunit (HLG70_19840-19880), chaperone protein (HLG70_26360-26365) and ribosomal protein (HLG70_21890-21935, HLG70_21970-22040; HLG70_22105-22190) were only found in the high concentration of Sb(III) comparative group (SSb0_5vsWSb0_5) (Figure 4).

### 2.8. SMAs-55 Displayed Reduced Intracellular Sb(III) Uptake

**Figure 5:**
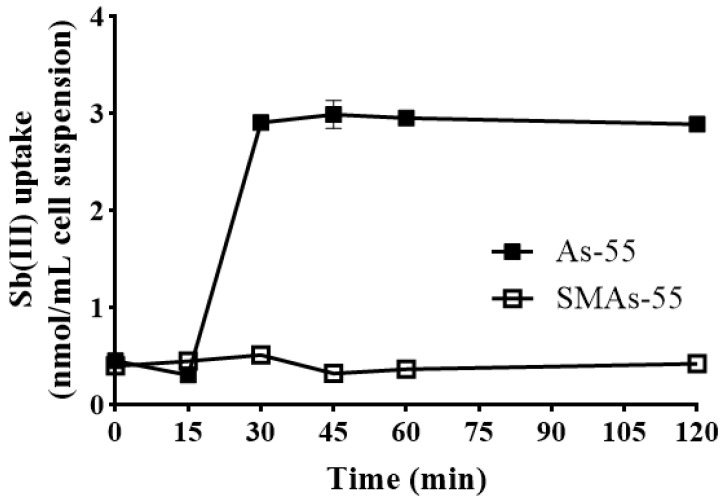
Intracellular Sb(III) uptake of As-55 and SMAs-55 when exposed to 50 µM Sb(III). The data are means of three replicates.

To further reveal the mechanism of spontaneous mutant SMAs-55 with higher resistance to Sb(III), intracellular Sb(III) uptake of both As-55 and SMAs-55 were determined. As shown in Figure 5, with 50 µm Sb(III) addition, the intracellular Sb(III) content in the wild-type strain As-55 increased with time, but the intracellular Sb(III) content in SMAs-55 remained at a low level, indicating that blocking the entry of Sb(III) into the cell is an effective strategy for SMAs-55 to obtain higher resistance to Sb(III). This is also consistent with the transcriptome analysis mentioned above, where the stress response of SMAs-55 to Sb(III) was lower than that of As-55.

## 3. Discussion

In this study, an As(III) oxidizing bacteria As-55 was isolated. As-55 showed high resistance to As(III) (MIC 40 mM), but low resistance to Sb(III) (MIC 0.5 mM). Later, we found that As-55 exhibited the ability to adapt to Sb(III). Since As-55 carried the classic *ars* operon which predicted to confer resistance to Sb(III). The relationship of As(III) resistance, oxidation and Sb(III) resistance of As-55 is worth exploring. Interestingly, we obtained an spontaneous mutant of As-55 under Sb(III) exposure, which we renamed SMAs-55. The Sb(III) resistance of SMAs-55 was significantly increased, while the As(III) ability was lost. To unveil the underlying mechanism, genomic and transcriptomic comparisons between spontaneous mutant SMAs-55 and wild-type As-55 were conducted.

Genomic analysis results showed that SMAs-55 deleted a whole gene isolated containing *aioXRSABCD*, *arsROC*-*mfs*-*arsH*, and *pstSCAB* operon, indicating that these genes are not necessary for As-55 to detoxify Sb(III), although their expression was upregulated under Sb(III) stress (Supplementary Figure S6). By losing genes encoding high-energy consuming functions, SMAs-55 could adapt to extremely environment quickly. This is consistent with the theory that genome streamlining is a universal mechanism for bacterial environmental adaptation [36,37]. Another deletion of SMAs-55 is a ribosomal RNA operon *rrn*. In recent years, researchers have pointed out that the *rrn* copy number is able to predict traits associated with resource availability [38], providing new insights for a comprehensive understanding of bacterial adaptation in natural habitats. Although the maximum reproductive rate of bacteria often increases with an increase in the *rrn* copy number, with all other conditions being the same, communities dominated by low *rrn* copy number bacteria have a higher carbon source utilization efficiency (CUE) than communities dominated by high *rrn* copy number bacteria [38]. Therefore, by deleting the ribosomal RNA operon *rrn*, the resource utilization rate is predicted to be improved to cope with environmental Sb(III) stress.

Transcriptomic comparison results showed that gene clusters encoding functions involved in cell wall and capsule biosynthesis, and gene clusters encoding CsbD family proteins were highly expressed in SMAs-55. CsbD is a general stress response protein that has been shown to be widely involved in phosphate starvation, heat, acid, and salt stress, as well as oxidative stress responses [39], but its exact functional role is still unclear. Its expression was increased in five different comparative groups, which may be a global stress response strategy for *Achromobacter* to respond to environmental changes.

Polysaccharides are mainly responsible for the surface characteristics of most bacteria and are present in different forms. Some are discharged polymers that only maintain limited binding to the cell surface, commonly referred to as extracellular polysaccharides (EPS) or mucopolysaccharides [40]. In contrast, other discrete surface layers closely tied to the cell surface are commonly referred to as capsule polysaccharides (CPS) [40]. Many bacteria produce abundant long-chain capsule polysaccharides, which are able to maintain strong binding states and form capsule structures that encapsulate cells or take the form of extracellular polysaccharides that are mainly secreted into the environment. These polymers have been shown to provide protection for these bacteria from various physical, chemical, and biological pressures, support biofilm frameworks, and play a crucial role in the interactions between bacteria and the environment [41]. Usually, the bacterial capsule is an important virulence factor for many pathogenic bacteria, which can protect bacteria from phagocytosis and being killed by serum factors, promoting bacterial colonization and biofilm formation at the infected site [42]. In this study, the entire capsule polysaccharide synthesis operon was upregulated in five comparative groups, indicating that increasing the production of capsule polysaccharides is an extremely effective strategy for *Achromobacter* to obtain increased antimony resistance and adapt to antimony-contaminated environments, which is consistent with the more mucoid phenotype of the spontaneous mutant SMAs-55. The lower intracellular Sb(III) uptake further verified the important role of capsule for SMAs-55 dealing with Sb(III) exposure. Moreover, the gene encoding TonB is also part of the genomic island that was deleted. The lack of this TonB protein and subsequent lack of possible TonB dependent receptors might also lead to decreases uptake of Sb(III).

## 4. Materials and Methods

### 4.1. Chemicals

All chemicals used in this study were of analytical grade. The As(III), Sb(III), Cu(II) and Cd(II) stock solutions were prepared using NaAsO_2_, C_8_H_4_K_2_O_12_Sb_2_·3H_2_O, CuSO_4_·5H_2_O and CdCl_2_·2/5H_2_O. DNA extraction kit (TIANamp Bacteria DNA Kit) was obtained from TIANGEN Biotech (Beijing, China), 2X SanTaq PCR Mix was purchased from Sangon Biotech (Shanghai, China). *TransZol* Up Plus RNA Kit, *TransStart*^®^ Green qPCR SuperMix and *EasyScript*^®^ One-Step gDNA Removal and cDNA Synthesis SuperMix were obtained from TransGen Biotech (Beijing, China).

### 4.2. Enrichment of As(III)/Sb(III) Resistant Bacteria and Screening for As(III) Oxidizing Bacteria

Samples from an As-/Sb-contaminated maize and chili farm adjacent to the Xikuangshan antimony mine, and mining area soil and mud were collected in Hunan Province, China. Enrichment experiments of As(III) or Sb(III) were performed as described in a previous study [43]. Briefly, the sample suspensions were cultured in mineral/R2A/TY medium containing 0.5 mM potassium antimonyl tartrate or sodium arsenite at 28 °C for 24 h. The mixture was then sub-cultured in a fresh corresponding medium with doubled As(III) or Sb(III) addition. After several rounds of subculturing, the enrichments were diluted and plated onto a corresponding medium containing As(III) or Sb(III) to select resistant isolates. Abiotic controls were set up for each culture medium containing no bacteria but with a corresponding concentration of As(Ⅲ) or Sb(III). The obtained pure As(III)/Sb(III)-resistant bacteria were further screened and analyzed for their As(III) oxidation ability using the silver nitrate method [44].

### 4.3. Phylogenetic Analysis of As-55

An isolate exhibiting As(III) oxidation, designated as strain As-55, was characterized and sequenced. 16S rRNA genes were amplified from genomic DNA extracted from strain As-55 using the primers of 27F and 1492R. The amplified fragment was ligated with pMD19-T vector and transformed into competent cell *E. coli* DH5α. A positive clone was sequenced with 3730xl DNA Analyzer (Applied Biosystems, Foster City, CA, USA) by BioSune Biotechnology Co., Ltd. (Fuzhou, China). The sequence was confirmed by BLAST in NCBI (https://blast.ncbi.nlm.nih.gov/Blast.cgi (accessed on 24 June 2024)) [45] and EzBioCloud (https://www.ezbiocloud.net/ (accessed on 24 June 2024)). The 16S and whole genome-based phylogenetic tree were constructed using the free bioinformatics platform Type (Strain) Genome Server [46] (https://tygs.dsmz.de (accessed on 24 June 2024)). The average nucleotide identity (ANI) values were calculated using the online tool on the Eztaxon website (https://www.ezbiocloud.net/tools/ani (accessed on 24 June 2024)).

### 4.4. Characterization of As-55

The cell morphology of As-55 was observed by scanning electron microscopy (SEM), as in a previous study [46]. Chemotaxis was determined using the 0.3% R2A agar plate [47] with and without arsenic. The As(III) oxidation assay was performed by selecting four bacteria of the same *Achromobacter* genus as the positive control and for comparison, the value of OD_600_ of strains were uniformed before being spotted onto medium containing different concentrations of As(Ⅲ), and then tested using the aforementioned silver nitrate method [44]. For the qualitative evaluation of As(III) oxidation, strain As-55 was precultured in R2A medium at 28 °C overnight under agitation at 180 rpm, and thereafter diluted at a ratio of 1:100 using fresh R2A with As(III) added at concentrations of 0, 10, 20, and 30 mM, respectively. Samples were harvested at different time intervals to assess As(V) production using the molybdate method [48]. The corresponding timepoint of the growth curve was determined based on the OD_600_ value versus time. For induction experiments, strain As-55 was precultured in R2A medium for overnight with and without 1 mM As(III), and the OD_600_ values of precultures were uniformed before being inoculated into fresh R2A medium with an increasing concentration of As(III). The treatment of a preculture with 1 mM As(III) exposure was used for induction. For metal(loid)s resistance, the growth curve of As-55 was monitored in presence of metal(loid)s (0.2, 2 mM As(III); 0.05, 0.1, 0.2 As(V); 0.05, 0.1 mM Roxarsone; 0.05, 0.1 mM Sb(III); 0.2, 2 mM Sb(V); 0.05, 0.1 mM Cu(II) and 0.05, 0.1 mM Cd(II)). The growth of cultures was determined at different intervals by following the absorbance at 600 nm.

### 4.5. Selection of Highly Sb(III) Resistance Spontaneous Mutant of As-55

Strain As-55 was inoculated in liquid R2A medium containing 2 mM Sb(III) (a concentration which much higher than the original minimum inhibitory concentration of As-55), and incubated at 28 °C with shaking at 180 rpm for 1 week for bacterial adaptation. After adaptation and subsequent growth, the culture was then streaked on Sb(III)-containing (a concentration where wild-type strain As-55 was not able to grow) medium to obtain single clones. Single clones were picked and streaked at least three times to obtain pure culture. 16S rRNA gene of clones with increased Sb resistance were sequenced and aligned with wild-type strain As-55 to ensure the presence of the correct, non-contaminated strain. One mucid clone obtained by Sb(III) selection was designated as strain SMAs-55 (spontaneous mutant of wild-type As-55).

### 4.6. Characterization and Comparative Analysis of As-55 and SMAs-55

Morphological changes in the wild-type strain As-55 and in its spontaneous mutant derivative SMAs-55 were observed by streaking them on solid LB plates and incubating them at 28 °C for 2 days before observation and taking pictures. Changes in arsenite oxidation were determined using the silver nitrate method [44]. The enzyme activity was assessed using the Mérieux Vitek 2 Compact detection system (Biomérieux, Marcy-l’Étoile, France). Changes in arsenic/antimony resistance were determined using an automatic growth curve analyzer Bioscreen C (Bioscreen, Helsinki, Finland). Briefly, overnight cultures of As-55 and SMAs-55 were diluted 1:100 in fresh R2A liquid medium containing different concentrations of As(III) (10, 20, 30, 40, and 50 mM) or Sb(III) (0.05, 0.5, 1 and 4 mM) and without metalloids. The OD_600_ value was measured every 1 h for up to 4 days.

### 4.7. Genomic and Transcriptomic Sequencing of As-55 and SMAs-55

The cultures of As-55 and SMAs-55 during the logarithmic phase were harvested using centrifugation at 7000 rpm for 3 min. The collected cells were washed with Phosphate-buffer solution (PBS) for three times to remove the residual culture medium and immediately frozen in liquid nitrogen for subsequent genome sequencing. Genome sequencing was performed by BioMarker Biotechnology Co., Ltd. (Beijing, China) using Nanopore PromethION platform (Oxford Nanopore Technologies, Oxford, UK). Briefly, total DNA of strain As-55 and SMAs-55 were extracted using the SDS method [49]. Then, Nanodrop (Thermo Fisher Scientific, Waltham, MA, USA), Qubit (Thermo Fisher Scientific, Waltham, MA, USA), and 0.35% agarose gel electrophoresis were used to assay the purity, concentration and integrity of the extracted genomic DNA. Sequencing libraries was constructed using SQK-LSK109 kit (Oxford Nanopore Technologies, Oxford, UK). The complete genome sequence was assembled using Canu v1.5 [50] software and annotated using RAST (RAST Server—RAST Annotation Server, https://rast.nmpdr.org/, accessed on 24 June 2024) [51]. Comparative genomic analysis was performed using MUMmer (v4.0.0) and LASTZ tools (v1.04.41) and Geneious prime 2022.1.1. (https://www.geneious.com (accessed on 24 June 2024)). The genomic data of As-55 and SMAs-55 are available on NCBI under accession number of CP074375.1 and CP090381.1, respectively.

For transcriptomic sequencing, 1% (*v*/*v*) of an overnight culture was inoculated into fresh R2A liquid medium and incubated at 28 °C with shaking at 180 rpm. The OD_600_ values of the cultures were monitored until they reached 0.6, at that time, As(III) and Sb (III) were added into the respective culture to obtain concentrations of 0.2, 2 mM As (III) and 0.05, 0.5 mM Sb (III), respectively. A metal-free culture was used as control. After exposure for 2 h, the cultures were collected by centrifugation at 12,000 rpm at 4 °C, the supernatant was removed, and the pellet was immediately frozen in liquid nitrogen and stored at −80 °C for later transcriptome sequencing. Transcriptomic sequencing was carried out at Allwegene Technology Co., Ltd. (Beijing, China). using Illumina PE150 sequencing platform (Illumina, San Diego, CA, USA). Bowtie2 v 2.3.5.1 was used to perform alignment analysis and expression level was quantified by FPKM (Fragments per kilobase of exon model per million mapped reads). DEGseq (v1.24.0) was used to identify differentially expressed genes as the standard of padj (qvalue) < 0.05. The sample codes were the control without metal stress of As-55 (WCK) and SMAs-55 (SCK); 0.2 mM As(III) stress treatment of As-55 (WAs0_2) and SMAs-55 (SAs0_2); 2 mM As(III) stress treatment of As-55 (WAs2) and SMAs-55 (SAs2); 0.05 mM Sb(III) exposure treatment of As-55 (WSb0_05) and SMAs-55 (SSb0_05); 0.5 mM Sb(III) stress treatment of As-55 (WSb0_5) and SMAs-55 (SSb0_5), with three biological replicates for each treatment. All transcriptomic data are available on NCBI under accession number PRJNA1150134.

### 4.8. Intracellular Antimony Determination

To determine the in vivo Sb(III) accumulation, wild-type strain As-55 and SMAs-55 were grown at 28 °C in R2A liquid medium and cells were harvested by centrifugation at 7000 rpm for 4 min. The collected cells were washed and resuspended in buffer A (75 mM HEPES-KOH, pH 7.5, 0.15 M KCl, and 1 mM MgSO_4_) [30]. To initiate the transport reaction, 50 µM Sb(III) were added to 1 mL of the cell suspension. Portions of 0.1 mL mixture were withdrawn at different timepoints (0, 15, 30, 45, 60, and 120 min) and washed twice with buffer A. These pellets were mineralized with 0.3 mL of HNO_3_ (68–70%) overnight at room temperature. Dissolved cells were incubated in a water bath at 70 °C for 10 min and diluted with sterile water to produce a final HNO_3_ concentration of 2%, and then were filtered through 0.22 µm filters before concentration determination. Sb(III) uptake by cells was determined by ICP-MS (NexIon 300 X, PerkinElmer, Waltham, MA, USA).

## 5. Conclusions

As(III)-oxidizing bacteria can convert toxic As(III) to less toxic As(V) and are widespread in nature, thus playing an essential role in environmental As bioremediation [20,32,52]. Many As(III)-oxidizers have been studied in detail [1,15,22,24] with the majority belonging to the phylum *Proteobacteria*. In this study, a strain As-55 possessing high As(III) resistance and oxidation ability, was isolated from a corn farm soil near an antimony mine. Interestingly, we obtained a spontaneous mutant SMAs-55 of the wild-type As-55 upon exposure to Sb(III). The mutant SMAs-55 displayed substantial increased resistance to Sb(III) by losing a gene island-encoding functions correlated to As(III) oxidation, specific phosphate transport, and resistance to various arsenic compounds, indicating a different detoxification mechanism under high Sb exposure. Here, we provided the evidence that efflux pumps are the key points for bacterial survival in extreme environments containing high Sb levels, and As(III) oxidase is an energy-generating enzyme that is only carried by bacteria at a concentration that is not excessively toxic. The ArsAB ATPase has been shown to be the most powerful As(III)/Sb(III) efflux pump to pump out excess Sb(III) from the cytoplasm. Moreover, a further increase in the Sb(III) resistance could be achieved by reducing the uptake of Sb(III).

## Data Availability

All data generated or analyzed during this study are included in the main text and its Supplementary Files. The DNA sequencing data of As-55 and SMAs-55 have been deposited in the NCBI database under accession numbers (GeneBank accession no: CP074375.1 for As-55 and CP090381.1 for SMAs-55). The RNA-seq data of As-55 and SMAs-55 have been deposited in NCBI database with accession number (SRA accession no: PRJNA1150134).

## Supplementary Materials

The following supporting information can be downloaded at: https://www.mdpi.com/article/10.3390/ijms26010107/s1.
