# Supplementary material for: Genome Deletions and Rewiring of the Transcriptome Underlying High Antimonite Resistance in Achromobacter sp. SMAs-55
Source: Int J Mol Sci. 2024 Dec 26;26(1):107. doi: 10.3390/ijms26010107 (PMC11719878; doi:10.3390/ijms26010107)
Supplement: Supplementary file 1 [file ijms-26-00107-s001.zip › ijms-3305253-SI.pdf]

Genome deletions and rewiring of the transcriptome underlie high antimonite resistance in *Achromobacter* sp. SMAs-55

Yanshuang Yu[1,2], Martin Herzberg[3], Aurora M. Pat-Espadas[4], Pablo Vinuesa[5], Renwei Feng[1], Barry Rosen[6], Seigo Amachi[7], Xianbo Jia[2], Christopher Rensing[1,*], Shungui Zhou[1]

1 College of Resources and Environment, Fujian Agriculture and Forestry University, Fuzhou, Fujian 350002, China

2 Institute of Resources, Environment and Soil Fertilizer, Fujian Academy of Agricultural Sciences

3 Department of Solar Materials Biotechnology (SOMA), Helmholtz Centre for Environmental Research – UFZ, 04318 Leipzig, Germany, EU

4 CONAHCYT-Institute of Geology, Estación Regional del Noroeste, Universidad Nacional Autónoma de México, Luis Donaldo Colosio s/n, Hermosillo, Sonora, Mexico

5 Centro de Ciencias Genómicas, Universidad Nacional Autónoma de México, Cuernavaca, Morelos, Mexico

6 Department of Cellular Biology and Pharmacology, Herbert Wertheim College of Medicine, Florida International University, Miami, FL, 33199, United States

7 Graduate School of Horticulture, Chiba University, Matsudo, Japan

* Correspondence: rensing@iue.ac.cn

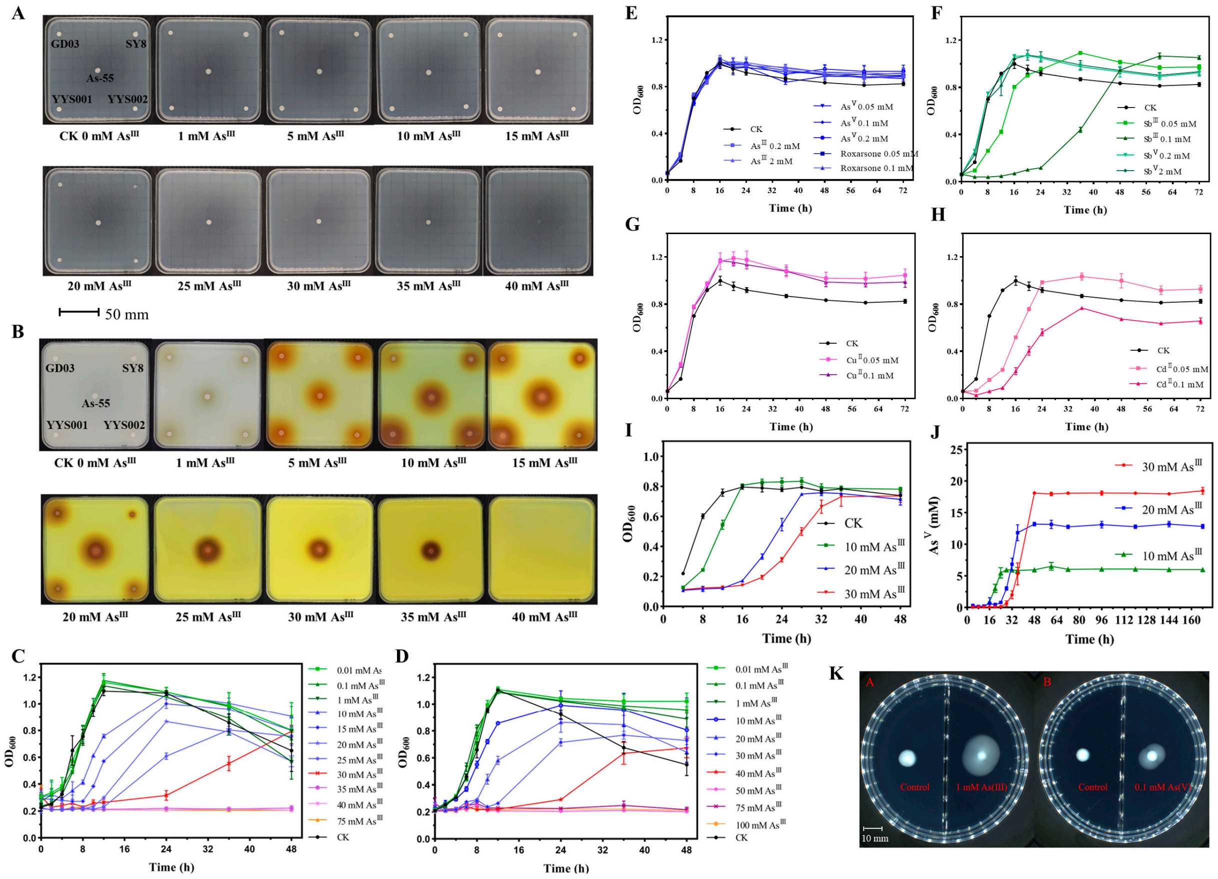

Fig. S1 Characterization of As-55. Growth (A) and arsenite oxidation (B) under different As(III) concentrations in As-55 compared to four other arsenite oxidizer of the genera *Achromobacter*. Growth curve of As-55 under different concentrations of As(III) with (D) and without (C) As induction. Growth curves of As-55 under low concentrations of As (E), Sb (F), Cu (G), and Cd (H). Arsenite oxidation (J) and differing growth condition (I) of As-55 under 0, 10, 20 and 30 mM As(III) exposure. Chemotaxis of As-55 to As (K). As-55 grow on medium with and without As, the left is R2A medium without As, while the right part is R2A medium containing As.

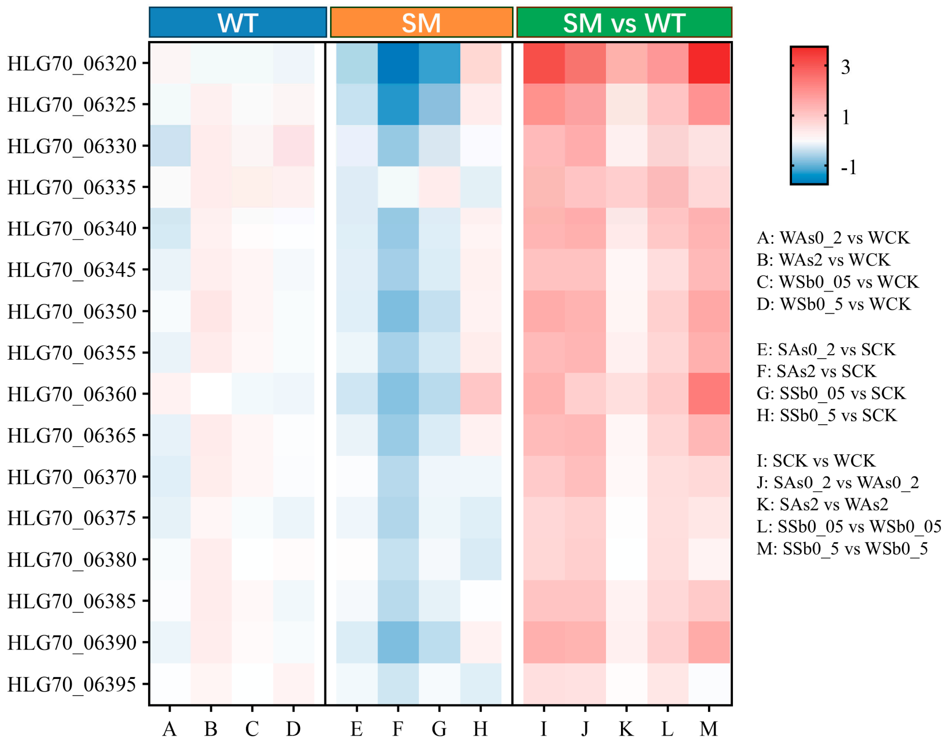

Fig. S2 Expression level of extracellular polymers biosynthesis related genes. Extracellular polymers biosynthesis related genes were more expressed in the spontaneous mutant (SM) compared to the wild type (WT).

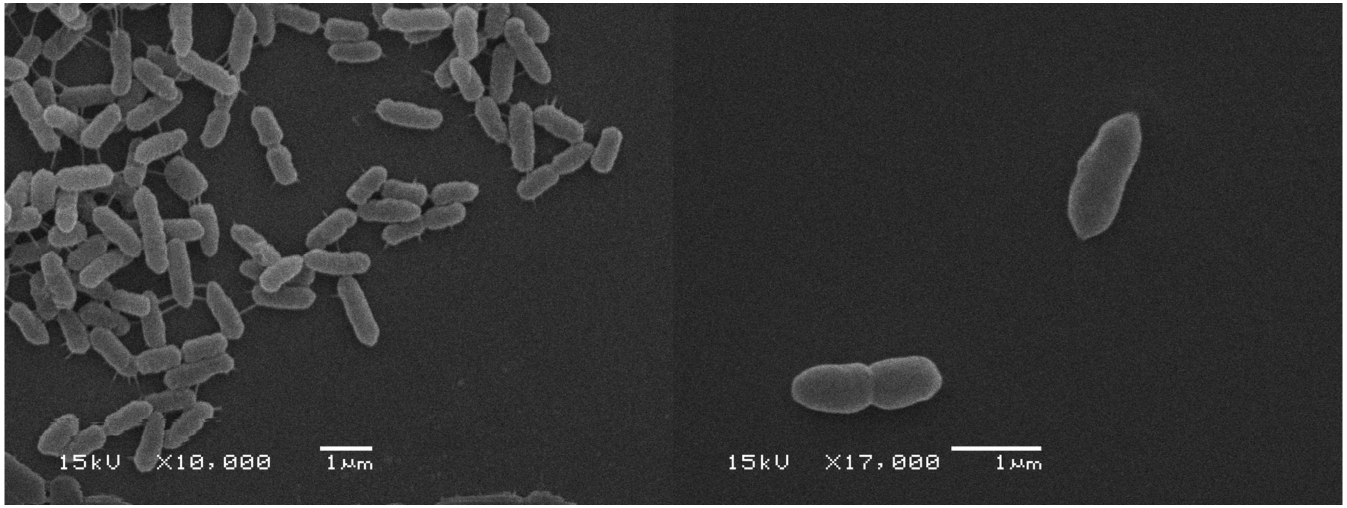

Fig. S3 Scanning electron microscope (SEM) images of *Achromobacter* sp. As-55

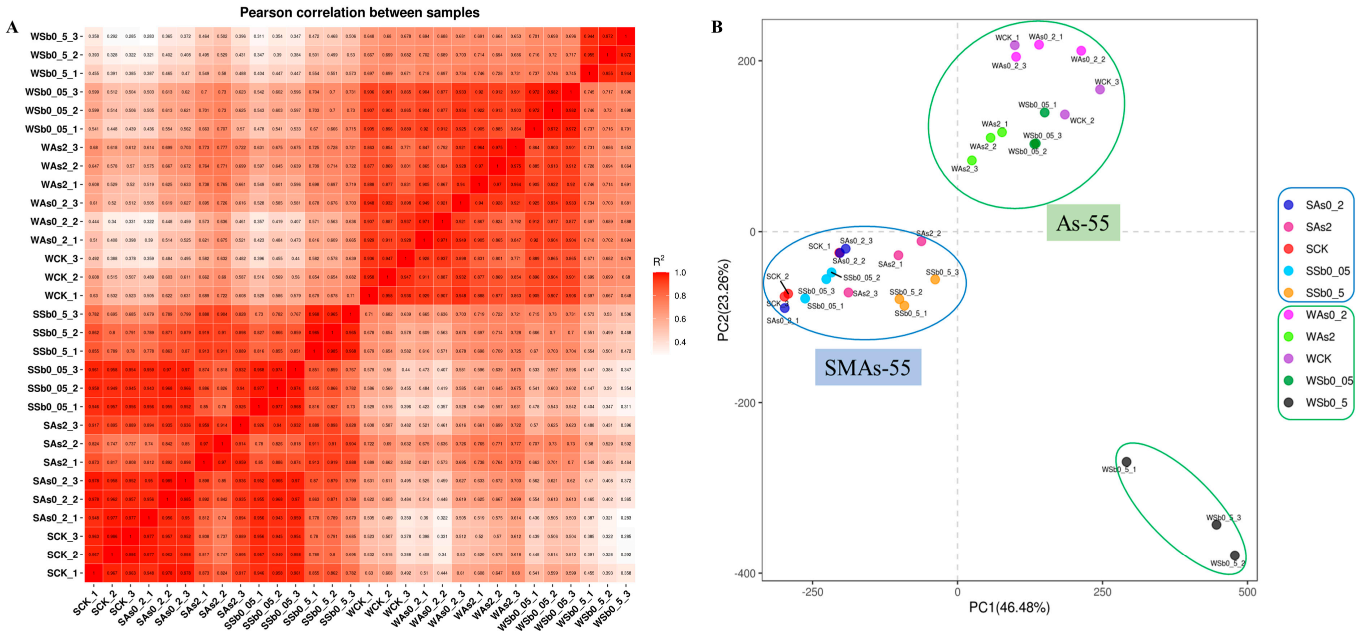

Fig. S4 Pearson correlation analysis (A) and PCA analysis (B) of strain As-55 and SMAs-55 with and without As/Sb stress. WCK: wild type strain As-55 without As/Sb stress (control group), WAs0_2/2: As-55 under the stress of 0.2/2 mM As(III), WSb0._05/0.5: As-55 under the stress of 0.05/0.5 mM Sb(III), SCK: spontaneous mutant SMAs-55 without As/Sb stress (control group), SAs0_2/2: SMAs-55 under the stress of 0.2/2 mM As(III), SSb0_05/0.5: SMAs-55 under the stress of 0.05/0.5 mM Sb(III). The person correlation coefficients within each sample group are all > 0.9, indicating a good biological replicability and parallelism of the samples. The first and second principal components of the PCA principal component analysis with explaining 46.48% and 23.26% of the sample variance, respectively. The distance between the treatment and control group of As-55 and SMAs-55 is relatively far, indicating that there is a certain difference in gene expression between the two bacteria under the same conditions.

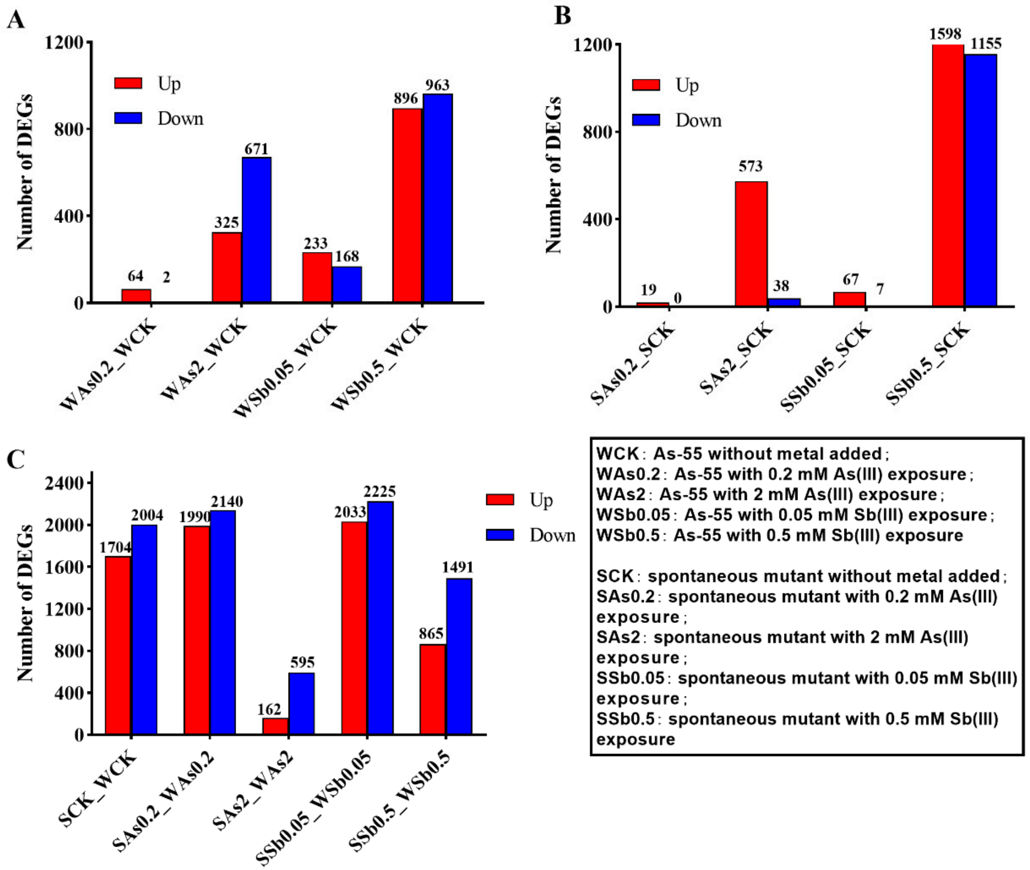

Fig. S5 Total transcriptomic information of As-55 and SMAs-55. DEGs of As-55 under As(III)/Sb(III) treatment compared to control treatment with no metal added in As-55 (A), DEGs of SMAs-55 with under As(III)/Sb(III) treatment compared to control treatment with no metal added in SMAs-55 (B), DEGs of As-55 compared to SMAs-55 with and without As(III)/Sb(III) exposure (C).

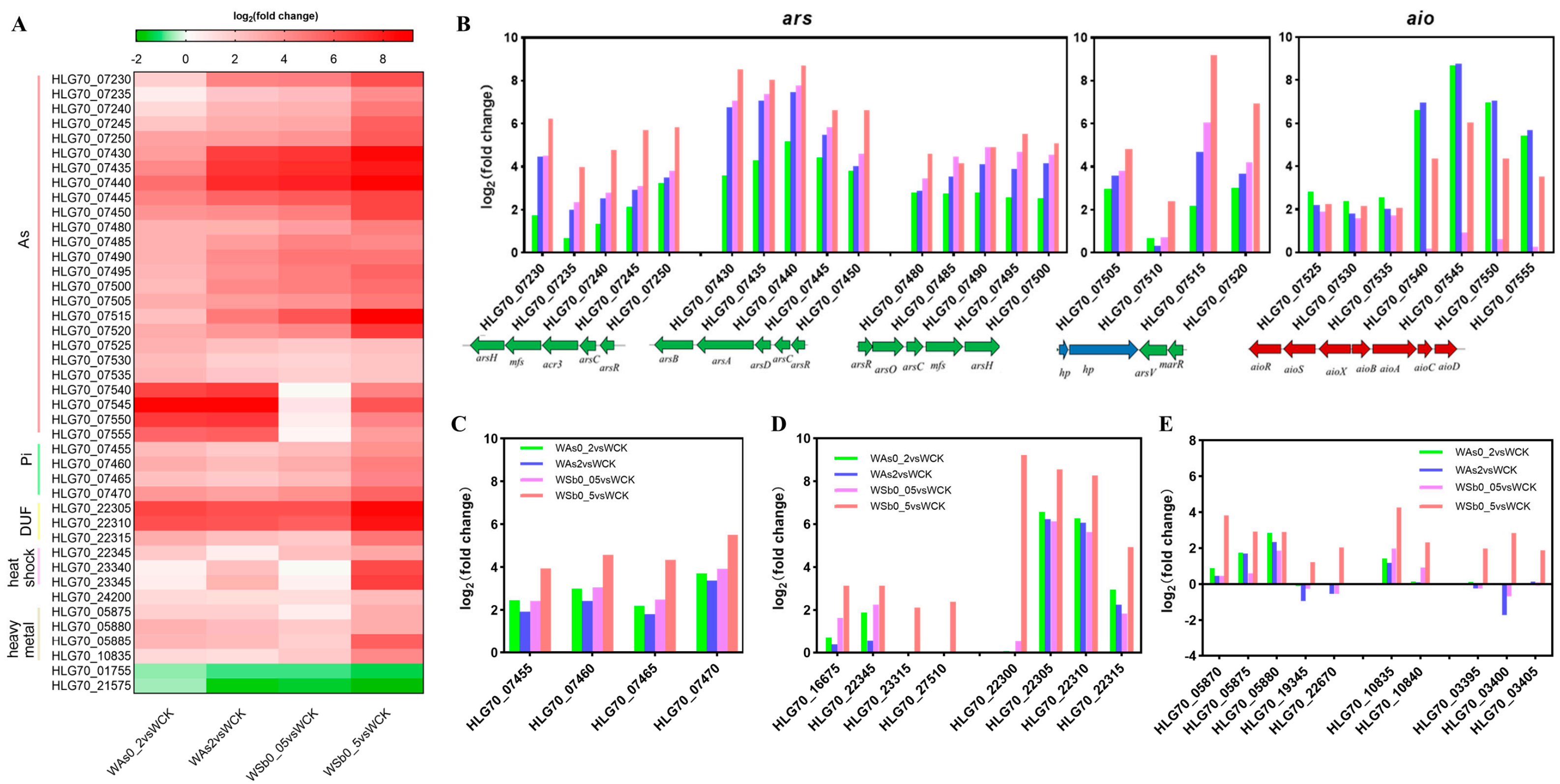

Fig. S6 General expression of genes in As-55 under treatment of exposure to differing concentrations of As and Sb, with genes encoding proteins correlated to As, Pi metabolism, heavy metal resistance and unknown functions (A). Genes on *ars*, *aio* operons and the intergenic regions were all upregulated under As and Sb exposure (B). Pi transport (C), DUF4148 domain proteins (D) and heavy metal resistance encoding genes (E) in As-55 were also upregulated under As and Sb exposure.

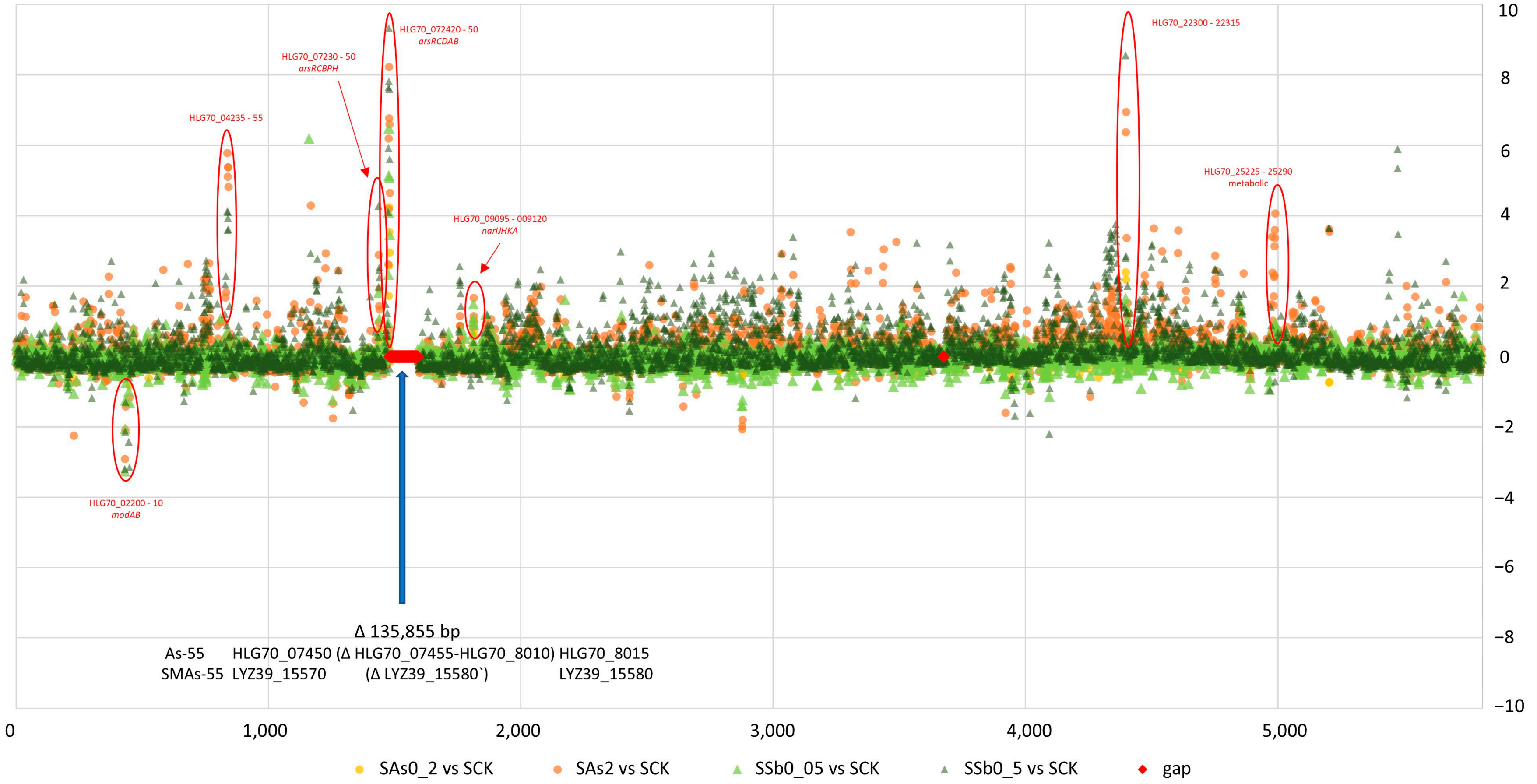

Fig. S7 Global transcriptome-level changes of SMAs-55 following As(III)/Sb(III) treatment. The expression levels of all genes of SMAs-55 treated with 0.2 mM (yellow) and 2 mM (orange) As(III) or 0.05 mM (light green) and 0.5 mM (dark green) Sb(III) when compared to the control with no As(III)/Sb(III) addition are given as log_2 (fold change) based on fragments per kilobase per million (FPKM) values.

Table S1 Meriere enzyme activity test

Number	Enzyme	As-55	SMAs-55	Number	Enzyme	As-55	SMAs-55
2	APPA	-	-	33	SAC	-	-
3	ADO	-	-	34	dTAG	-	-
4	PyrA	+	+	35	dTRE	-	-
5	IARL	-	-	36	CIT	+	-
7	dCEL	-	-	37	MNT	-	-
9	BGAL	-	-	39	5KG	-	-
10	H2S	-	-	40	ILATk	+	+
11	BANG	-	-	41	AGLU	-	-
12	AGLTp	-	+	42	SUCT	+	+
13	dGLU	-	-	43	NAGA	-	-
14	GGT	-	-	44	AGAL	-	-
15	OFF	-	-	45	PHOS	-	-
17	BGLU	-	-	46	GlyA	-	-
18	dMAL	-	-	47	ODC	-	-
19	dMAN	-	-	48	LDC	-	-
20	dMNE	-	-	53	IHISa	-	-
21	BXYL	-	-	56	XMT	-	-

Number	Enzyme	As-55	SMAs-55	Number	Enzyme	As-55	SMAs-55
22	BAlap	-	-	57	BGUR	-	-
23	ProA	+	+	58	O129R	-	-
26	LIP	-	-	59	GGAA	-	-
27	PLE	-	-	61	IMLTa	+	-
29	TryA	+	+	62	ELLM	+	+
31	URE	-	-	64	ILATa	-	-
32	dSOR	-	-				

Table S2 Genomic comparison of wild type strain As-55 and spontaneous mutant SMAs-55

Target	Start target	End target	Query	Start query	End target	type	Length target
As-55	1644870	1780794	SMAs-55	3381927	3381927	Deletion	135925
As-55	2749817	2750224	SMAs-55	4350949	4350949	Deletion	408
As-55	2750324	2750731	SMAs-55	4351048	4351048	Deletion	408
As-55	4018110	4024103	SMAs-55	5618426	5618426	Deletion	5994

Table S3 Transcriptomic data of wild type strain As-55 and spontaneous mutant SMAs-55

locus_tag	gene	product	1	2	3	4	5	6	7	8	9	10	11	12	13
**As/Sb related**															
***ars*1-cluster**															
HLG70_07230	*arsH*	arsenical resistance protein ArsH	1.71	4.46	4.52	6.22	0.55	2.89	1.82	4.29	0.6	-0.51	-0.97	-2.16	-1.52
HLG70_07235	*mfs*	MFS transporter	0.68	2.01	2.37	3.97	0.19	0.97	0.59	1.99	0.78	0.34	-0.26	-1.06	-1.38
HLG70_07240	*arsB*	ACR3 family arsenite efflux transporter	1.33	2.53	2.81	4.78	0.33	1.44	0.71	2.44	0.79	-0.17	-0.31	-1.38	-1.74
HLG70_07245	*arsC*	arsenate reductase ArsC	2.12	2.92	3.09	5.7	0.53	1.35	0.67	2.24	0.55	-1	-1.03	-1.93	-3.11
HLG70_07250	*arsR*	helix-turn-helix transcriptional regulator	3.25	3.48	3.82	5.81	0.78	1.93	1.08	2.53	0.64	-1.78	-0.92	-2.16	-2.84
***ars*2-cluster**															
HLG70_07430	*arsB*	arsenic transporter	3.59	6.76	7.07	8.53	1.73	6.2	4.1	7.65	-0.47	-2.28	-1.03	-3.5	-1.55
HLG70_07435	*arsA*	arsenical pump-driving ATPase	4.28	7.07	7.38	8.05	2.59	6.78	5.15	7.82	-1.24	-2.88	-1.53	-3.54	-1.65
HLG70_07440	*arsD*	arsenite efflux transporter metallochaperone	5.16	7.47	7.75	8.72	4.25	8.24	6.49	9.33	-2.2	-3.07	-1.44	-3.53	-1.77
HLG70_07445	*arsC*	arsenate reductase ArsC	4.4	5.46	5.85	6.62	3.54	6.61	5.08	7.61	-2.47	-3.29	-1.32	-3.3	-1.67
HLG70_07450	*arsR*	helix-turn-helix transcriptional regulator	3.8	4.02	4.62	6.61	2.96	4.66	3.47	5.61	-2.22	-3.01	-1.59	-3.42	-3.4
***ars*3-cluster**															
HLG70_07475	*arsR*	helix-turn-helix transcriptional regulator	0.68	0.48	0.99	4.04	0	0	0	0	0	0	0	0	0
HLG70_07480	*arsR*	helix-turn-helix transcriptional regulator	2.8	2.86	3.44	4.61	0	0	0	0	0	0	0	0	0
HLG70_07485	*arsO*	NAD(P)/FAD-dependent oxidoreductase	2.75	3.56	4.45	4.15	0	0	0	0	0	0	0	0	0
HLG70_07490	*arsC*	arsenate reductase (glutaredoxin)	2.81	4.11	4.9	4.9	0	0	0	0	0	0	0	0	0
HLG70_07495	*mfs*	MFS transporter	2.58	3.89	4.68	5.53	0	0	0	0	0	0	0	0	0
HLG70_07500	*arsH*	arsenical resistance protein ArsH	2.51	4.17	4.55	5.09	0	0	0	0	0	0	0	0	0
HLG70_07505	*uspA*	universal stress protein	2.97	3.58	3.81	4.82	0	0	0	0	0	0	0	0	0
HLG70_07510	*hyp*	hypothetical protein	0.66	0.3	0.73	2.38	0	0	0	0	0	0	0	0	0

HLG70_07515	*arsV*	FAD-dependent oxidoreductase	2.17	4.69	6.04	9.19	0	0	0	0	0	0	0	0	0
HLG70_07520	*marR*	MarR family transcriptional regulator	3.01	3.66	4.2	6.94	0	0	0	0	0	0	0	0	0
***ars*4-cluster**															
HLG70_07525	*aioR*	sigma-54-dependent Fis family	2.8	2.21	1.88	2.23	0	0	0	0	0	0	0	0	0
HLG70_07530	*aioS*	HAMP domain-containing histidine kinase	2.39	1.79	1.57	2.15	0	0	0	0	0	0	0	0	0
HLG70_07535	*aioX*	PhnD/SsuA/transferrin family substrate-	2.53	2.01	1.72	2.06	0	0	0	0	0	0	0	0	0
HLG70_07540	*aioB*	arsenite oxidase small subunit	6.59	6.95	0.16	4.37	0	0	0	0	0	0	0	0	0
HLG70_07545	*aioA*	arsenite oxidase large subunit	8.69	8.79	0.92	6.01	0	0	0	0	0	0	0	0	0
HLG70_07550	*aioC*	c-type cytochrome	6.94	7.05	0.63	4.36	0	0	0	0	0	0	0	0	0
HLG70_07555	*aioD*	GTP 3',8-cyclase MoaA	5.42	5.67	0.28	3.51	0	0	0	0	0	0	0	0	0
**Pi transport**															
HLG70_07455	*pstB*	phosphate ABC transporter ATP-binding	2.45	1.93	2.43	3.94	0	0	0	0	0	0	0	0	0
HLG70_07460	*pstA*	phosphate ABC transporter permease PstA	2.99	2.42	3.05	4.57	0	0	0	0	0	0	0	0	0
HLG70_07465	*pstC*	phosphate ABC transporter permease	2.19	1.81	2.49	4.34	0	0	0	0	0	0	0	0	0
HLG70_07470	*pstS*	phosphate ABC transporter substrate-	3.7	3.38	3.93	5.51	0	0	0	0	0	0	0	0	0
**DUF4148**															
HLG70_16675		DUF4148 domain-containing protein	0.72	0.39	1.64	3.13	0.28	0.12	0.22	0.27	0.07	-0.32	-0.2	-1.41	-2.97
HLG70_22345		DUF4148 domain-containing protein	1.89	0.55	2.25	3.13	1.22	1.52	1.09	2.01	-1.5	-2.12	-0.53	-2.72	-2.81
HLG70_23315		DUF4148 domain-containing protein	-0.3	-0.4	-0.41	2.11	-0.01	0.08	0.14	0.04	-0.38	-0.05	0.09	0.11	-2.64
HLG70_27510		DUF4148 domain-containing protein	-0.32	-0.38	-0.3	2.38	0.02	-0.24	-0.04	-0.25	-0.15	0.24	-0.02	0.05	-2.97
HLG70_22300		mandelate racemase/muconate lactonizing	0.07	-0.5	0.54	9.21	0.09	0.63	0.49	8.56	-0.71	-0.65	0.41	-0.83	-1.55

		enzyme family protein													
HLG70_22305		DUF4148 domain-containing protein	6.57	6.23	6.14	8.55	2.4	6.38	1.66	2.8	0.44	-3.69	0.58	-4.12	-5.5
HLG70_22310		DUF4148 domain-containing protein	6.27	6.06	5.64	8.27	2.19	6.95	1.46	2.86	0.54	-3.5	1.42	-3.71	-5.07
HLG70_22315		TetR/AcrR family transcriptional regulator	2.95	2.25	1.84	4.93	0.58	3.38	0.35	1.26	-0.5	-2.82	0.63	-2.05	-4.37
**ROS-related antioxidation**															
HLG70_05885	*Gpx*	glutathione peroxidase	2.55	2.39	1.73	5.64	0.77	4.3	0.47	2.94	-0.73	-2.47	1.17	-2.05	-3.63
HLG70_16690		thioredoxin family protein	-0.21	1.42	0.39	2.02	-0.18	2.48	0.23	1.64	-0.91	-0.83	0.15	-1.13	-1.49
HLG70_23020		organic hydroperoxide resistance protein	0.16	-0.23	2.53	4.71	-0.16	2.99	0.08	1.84	-0.44	-0.72	2.78	-2.96	-3.5
HLG70_21670	*CaT*	catalase	0.16	-0.84	-0.42	1.7	-0.18	1.45	-0.38	1.79	-2.68	-2.97	-0.4	-2.7	-2.77
HLG70_27980	*PoD*	peroxiredoxin	0.47	-0.31	-0.17	1.03	-0.23	0.76	-0.44	0.95	-1.28	-1.94	-0.22	-1.62	-1.55
HLG70_05800		thioredoxin family protein	0.17	-0.58	-0.36	1.57	-0.49	0.77	-0.56	1.35	-1.72	-2.34	-0.37	-1.98	-2.13
HLG70_16830		thioredoxin family protein	-0.22	-0.08	-0.16	1.12	-0.08	-0.05	-0.02	-0.17	0.06	0.24	0.09	0.13	-1.42
HLG70_09755	*GST*	glutathione S-transferase N-terminal domain-containing protein	0.57	0.19	0.27	1.53	-0.04	1.38	0	1.61	-0.89	-1.46	0.3	-1.23	-1
HLG70_13065		oxidative damage protection protein	0.02	-0.58	-0.25	2.22	0.04	0.69	-0.02	0.98	-1.27	-1.2	0	-1.1	-2.7
HLG70_03275	*sodB*	superoxide dismutase [Fe]	0.22	-1.11	-0.13	0.74	-0.43	1.45	-0.5	2.14	-2.69	-3.3	-0.13	-3.13	-1.48
HLG70_15535		peroxiredoxin	0.26	-0.57	0.75	-0.05	-0.09	2.32	-0.13	3.4	-2.99	-3.29	-0.1	-3.93	0.27
HLG70_14305	*POD*	peroxiredoxin	-0.15	-1.38	-0.99	0.28	-0.17	0.53	-0.57	1.09	-3.15	-3.13	-1.25	-2.81	-2.53
**chaperone**															
HLG70_23340	*Hsp20*	Hsp20/alpha crystallin family protein	0.41	2.18	0.16	6.31	0.06	2.95	0.02	0.92	0.1	-0.2	0.87	-0.11	-5.48
HLG70_23345	*Hsp20*	Hsp20/alpha crystallin family protein	0.72	2.67	0.39	6.82	-0.04	3.59	0.18	1.42	-0.04	-0.76	0.88	-0.31	-5.63
HLG70_22675	*hslU*	ATP-dependent protease ATPase subunit	0.23	1.65	0.56	1.34	-0.06	2.31	0.13	1.57	0.22	-0.03	0.87	-0.28	0.26
HLG70_22680	*hslV*	ATP-dependent protease subunit HslV	0.08	1.23	0.4	1.88	-0.05	2.08	0	0.93	0.34	0.26	1.19	-0.11	-0.8
HLG70_16685	*dnaK*	molecular chaperone DnaK	0.34	2.24	0.8	3.99	-0.45	3.54	0.02	2.38	-1.1	-1.85	0.2	-1.95	-2.9

HLG70_14810	*hslO*	Hsp33 family molecular chaperone HslO	0.06	-0.32	0.01	2.14	-0.17	0.22	-0.11	0.67	-0.65	-0.84	-0.11	-0.83	-2.31
HLG70_16710	*hrcA*	heat-inducible transcriptional repressor	-0.3	-0.61	-0.38	1.59	-0.05	0.12	-0.02	0.14	-1.06	-0.77	-0.33	-0.76	-2.7
HLG70_03780	*lon*	endopeptidase La	0.37	0.22	0.89	2.2	-0.4	2.21	0.47	1.27	-1.68	-2.41	0.31	-2.16	-2.79
HLG70_02960	*clpB*	ATP-dependent chaperone ClpB	0.33	0.9	-0.01	1.96	-0.22	2.47	-0.26	0.86	-0.72	-1.23	0.84	-1.04	-2.02
HLG70_03770	*clpP*	ATP-dependent Clp endopeptidase proteolytic subunit ClpP	0.16	-0.59	0.21	1.33	-0.22	1.04	-0.3	1.96	-2.05	-2.38	-0.42	-2.62	-1.62
HLG70_03320	*clpA*	ATP-dependent Clp protease ATP-binding subunit ClpA	0.27	-0.77	-0.28	1.29	-0.42	0.47	-0.54	0.9	-1.97	-2.61	-0.72	-2.29	-2.55
HLG70_03740	*Csp*	cold-shock protein	-0.99	-1.82	-1.45	1.94	-0.31	1.08	-0.74	1.75	-3.74	-3.01	-0.85	-3.09	-4.12
HLG70_03750	*Csp*	cold-shock protein	-1.28	-3	-2.4	1.44	-0.43	-0.13	-0.8	0.64	-4.7	-3.8	-1.83	-3.16	-5.7
HLG70_10915	*Csp*	cold-shock protein	-0.89	-1.02	-0.47	1.29	0.02	-0.37	-0.25	-0.32	-0.3	0.66	0.35	-0.14	-2.11
HLG70_15860	*Csp*	cold-shock protein	-1.36	-1.91	-1.32	2.09	-0.12	0.4	0.34	0.48	-3.4	-2.11	-1.08	-1.79	-5.2
HLG70_26360	*groL*	chaperonin GroEL	0.25	2.14	1.45	-0.24	-0.73	3.62	0.29	3.66	-1.14	-2.07	0.34	-2.36	2.57
HLG70_26365	*groES*	co-chaperone GroES	0.28	2.22	1.41	0.25	-0.72	3.56	0.06	3.64	-0.95	-1.89	0.39	-2.36	2.26
HLG70_03765	*tig*	trigger factor	0.27	-0.3	0.67	-0.56	-0.22	0.7	-0.21	2.12	-1.17	-1.62	-0.18	-2.11	1.31
HLG70_03775	*clpX*	ATP-dependent Clp protease ATP-binding subunit ClpX	0.17	-0.82	-0.21	0.47	-0.48	0.54	-0.55	1.27	-1.93	-2.53	-0.57	-2.33	-1.32
**Fe-S cluster**															
HLG70_03805	*iscR*	Fe-S cluster assembly transcriptional regulator IscR	0.39	-0.42	-0.05	5.3	-0.28	2	-0.21	2.34	-1.65	-2.27	0.77	-1.87	-4.8
HLG70_03810	*iscS*	IscS subfamily cysteine desulfurase	0.57	-0.49	-0.03	4.79	-0.25	2.18	-0.46	2.56	-1.66	-2.44	1	-2.16	-4.09
HLG70_03815	*iscU*	Fe-S cluster assembly scaffold IscU	0.7	-0.64	-0.14	4.49	-0.43	2.25	-0.71	2.73	-1.94	-3.02	0.95	-2.57	-3.89
HLG70_03820	*iscA*	iron-sulfur cluster assembly protein IscA	0.56	-0.77	-0.33	4.5	-0.44	1.9	-0.64	2.45	-1.83	-2.78	0.85	-2.2	-4.07
HLG70_03825	*hscB*	Fe-S protein assembly co-chaperone HscB	0.6	-0.17	-0.02	4.22	-0.18	0.97	-0.22	1.58	-0.28	-1.01	0.85	-0.55	-3.12
HLG70_03830	*hscA*	Fe-S protein assembly chaperone HscA	0.33	-0.07	0.06	2.81	-0.11	0.06	-0.13	0.9	0.27	-0.12	0.41	0.02	-1.82

HLG70_03835	*fdx*	ISC system 2Fe-2S type ferredoxin	0.07	-1.07	-0.72	3.55	-0.28	0.61	0.15	1.35	-1.42	-1.72	0.27	-0.6	-3.81
HLG70_03840	*iscX*	Fe-S cluster assembly protein IscX	0.4	-0.91	-0.55	3.22	-0.48	0.18	0.23	0.87	-1.3	-2.13	-0.21	-0.59	-3.84
HLG70_15820	*ytfE*	iron-sulfur cluster repair protein YtfE	-0.11	0.27	-0.1	1.18	0.04	-0.06	0.13	-0.3	0.45	0.65	0.12	0.61	-1.22
HLG70_18765	*erpA*	iron-sulfur cluster insertion protein ErpA	0.2	-0.57	-0.37	1.75	-0.36	0.74	-0.72	1.04	-2.05	-2.56	-0.74	-2.46	-2.95
HLG70_20215		2Fe-2S iron-sulfur cluster binding domain-containing protein	-0.09	0.14	0.03	2.43	0.04	0.04	0.02	-0.04	0.32	0.49	0.22	0.24	-2.34
HLG70_20220		heme-binding protein	-0.05	0.25	0.07	1.53	0.11	0.17	0.23	0.09	0.43	0.64	0.34	0.52	-1.2
HLG70_04235		ABC transporter substrate-binding protein	0.36	3.4	1.03	4.82	0.41	5.79	0.72	4.13	0.68	0.78	3.07	0.3	-0.2
HLG70_04240		phosphonopyruvate decarboxylase	-0.11	2.58	0.33	4.04	0	5.12	0.37	3.59	0.39	0.55	2.94	0.37	-0.24
HLG70_04245		aldehyde dehydrogenase	0.07	1.99	-0.05	3.07	-0.08	5.39	-0.02	3.94	0.02	-0.08	3.43	-0.01	0.71
HLG70_04250		aldehyde dehydrogenase family protein	0.24	1.64	-0.02	3.33	0.01	5.38	0.06	4.11	-0.5	-0.69	3.24	-0.49	0.09
HLG70_04255		NAD(P)-dependent oxidoreductase	0.3	1.49	0.03	2.25	0	4.82	0.04	3.61	-0.24	-0.49	3.09	-0.29	0.93
**Heavy metal**															
HLG70_05870	*cueR*	Cu(I)-responsive transcriptional regulator	0.89	0.45	0.46	3.82	-0.04	0.5	0.15	0.98	-0.16	-1.04	-0.11	-0.53	-3.18
HLG70_05875	*copA*	copper-translocating P-type ATPase	1.75	1.7	0.6	2.94	0.15	0.93	-0.02	0.41	0.48	-1.07	-0.29	-0.2	-2.23
HLG70_05880	*copC*	heavy-metal-associated domain-containing protein	2.84	2.36	1.87	2.9	0.67	1.56	0.27	0.99	-0.68	-2.8	-1.47	-2.34	-2.78
HLG70_19345		copper resistance protein NlpE N-terminal domain-containing protein	0.02	-0.94	-0.26	1.23	-0.12	0.44	-0.27	0.67	-1.93	-2.02	-0.55	-2.01	-2.68
HLG70_22670		copper-binding protein	-0.07	-0.55	-0.54	2.04	-0.1	0.11	0	0.17	-0.99	-0.97	-0.33	-0.51	-3.05
HLG70_10835		heavy metal translocating P-type ATPase	1.43	1.19	1.98	4.26	0.36	1.62	0.72	2.19	-0.19	-1.22	0.24	-1.51	-2.45
HLG70_10840		helix-turn-helix domain-containing protein	0.14	0.09	0.92	2.32	-0.06	0.13	0.24	0.79	0.07	-0.09	0.11	-0.68	-1.65
HLG70_03395		TetR/AcrR family transcriptional regulator	0.12	-0.25	-0.25	1.98	0	-0.19	-0.15	-0.01	-0.35	-0.43	-0.3	-0.31	-2.53
HLG70_03400		XRE family transcriptional regulator	0.06	-1.72	-0.69	2.85	-0.33	-0.17	-0.28	-0.31	-2.85	-3.19	-1.3	-2.5	-6.19

HLG70_03405	*MntH*	Nramp family divalent metal transporter	0.05	0.14	0.09	1.88	-0.04	-0.28	-0.09	-0.2	0.5	0.45	0.08	0.26	-1.77
HLG70_16725	*fur*	ferric iron uptake transcriptional regulator	0.42	-0.88	-0.01	-0.52	-0.31	0.52	-0.36	1.15	-1.83	-2.52	-0.43	-2.25	-0.35
HLG70_16705		ferrochelatase	0	-0.67	-0.17	-0.29	-0.09	0.3	-0.06	1.07	-1.42	-1.47	-0.46	-1.37	-0.25
**S oxidation**															
HLG70_08835	*soxY*	thiosulfate oxidation carrier protein SoxY	0.19	-0.71	0.17	-1.34	-0.14	0.87	-0.15	2.18	-2.45	-2.73	-0.87	-2.84	0.89
HLG70_08840	*soxZ*	thiosulfate oxidation carrier complex protein SoxZ	0.06	-0.97	0	-1.26	-0.16	1.15	-0.18	2.57	-2.94	-3.11	-0.81	-3.19	0.71
HLG70_08845	*soxA*	sulfur oxidation c-type cytochrome SoxA	0.16	-0.67	-0.2	-1.6	-0.11	0.85	-0.23	2.03	-2.32	-2.54	-0.8	-2.42	1.12
HLG70_08850	*soxX*	sulfur oxidation c-type cytochrome SoxX	0.25	-0.52	-0.04	-1.86	-0.13	0.87	-0.23	2.12	-2.21	-2.55	-0.83	-2.47	1.57
HLG70_08855		TlpA family protein disulfide reductase	0.31	-0.52	0.06	-1.43	0.02	0.57	-0.08	1.79	-1.61	-1.86	-0.53	-1.81	1.41
HLG70_08860	*soxB*	thiosulfohydrolase SoxB	0.21	-0.27	0.25	-1.04	-0.05	0.33	-0.01	1.43	-0.92	-1.13	-0.32	-1.25	1.36
**N metabolism**															
HLG70_11415	*napE*	periplasmic nitrate reductase, NapE protein	-0.28	-1.44	-0.89	0.44	-0.29	0.34	-0.31	0.74	-2.94	-2.9	-1.16	-2.41	-2.82
HLG70_11420	*napD*	chaperone NapD	0.17	-0.92	-0.43	0.28	-0.38	0.21	-0.38	0.6	-2.11	-2.62	-0.99	-2.12	-1.99
HLG70_11425	*napA*	periplasmic nitrate reductase subunit alpha	0.11	-1.17	-0.61	-1.1	-0.36	0.14	-0.48	0.38	-2.53	-2.96	-1.22	-2.46	-1.24
HLG70_11430	*napB*	nitrate reductase cytochrome c-type subunit	0.02	-1.18	-0.76	-1.49	-0.39	-0.07	-0.65	0.27	-2.62	-2.99	-1.51	-2.57	-1.06
HLG70_09095	*narI*	respiratory nitrate reductase subunit gamma	0.37	0.32	1.87	0.3	0.18	0.07	0.41	-0.24	0.39	0.24	0.13	-1.12	-0.34
HLG70_09100	*narJ*	nitrate reductase molybdenum cofactor assembly chaperone	0.62	0.47	2.44	0	0.31	0.19	0.64	-0.27	0.27	0	-0.01	-1.59	-0.19
HLG70_09105	*narH*	nitrate reductase subunit beta	1.07	0.55	2.78	-0.64	0.59	0.84	1	0.04	-0.18	-0.62	0.1	-2.02	0.31
HLG70_09110	*narG*	nitrate reductase subunit alpha	0.78	0.1	2.1	-2.1	1.07	1.67	1.49	0.4	-1.28	-0.94	0.29	-1.95	1.03
HLG70_28160	*NasAB*	molybdopterin-dependent oxidoreductase	-0.02	0.44	0.2	0.26	0.08	-0.32	-0.03	-0.27	0.82	0.97	0.05	0.54	0.11
HLG70_09115	*narK*	NarK family nitrate/nitrite MFS transporter	0.91	0.13	2.14	-1.47	0.74	1.15	1.1	0.07	-0.64	-0.77	0.37	-1.75	0.71
HLG70_09120	*narK*	NarK/NasA family nitrate transporter	0.51	0.01	1.66	-1.18	0.58	1	0.9	0.1	-0.65	-0.54	0.34	-1.48	0.44
HLG70_11650	*nirK*	nitrite reductase, copper-containing	0.29	-0.63	-0.14	-1.8	-0.05	-0.15	-0.09	-0.47	-0.73	-1.01	-0.24	-0.74	0.42

HLG70_11665	*norBC*	nitric-oxide reductase large subunit	-0.12	0.4	0.15	0	0.1	-0.26	0.16	-0.26	0.7	0.96	0.03	0.64	0.24
HLG70_01385	*nosF*	ABC transporter ATP-binding protein	0.31	0.5	1.12	0.17	0.09	-0.11	0.08	-0.28	0.61	0.44	0	-0.49	-0.03
HLG70_01390	*nosD*	nitrous oxide reductase family maturation protein NosD	0.93	0.46	1.62	-0.2	0.12	0.09	0.16	-0.04	0.24	-0.52	-0.14	-1.28	0.21
HLG70_01395	*nosZ*	TAT-dependent nitrous-oxide reductase	1.54	0.38	2.12	-2.44	0.21	0.91	0.6	0.35	-1.51	-2.81	-0.99	-3.09	1.08
HLG70_01400	*nosR*	regulatory protein NosR	0.15	-0.48	-0.12	-1.42	0	-0.1	-0.07	-0.31	-0.53	-0.63	-0.16	-0.54	0.39
**NO detoxification**															
HLG70_11760	*hmpA*	NO-inducible flavohemoprotein	0.13	0.06	0.35	1.49	0	-0.15	-0.01	0.36	0.07	-0.01	-0.14	-0.35	-1.25
HLG70_15270		ferredoxin family protein	-0.62	-1.14	-0.64	2.36	-0.28	0.79	0.02	1.32	-2.49	-2.1	-0.56	-1.9	-3.72
HLG70_15275		ferredoxin--NADP reductase	0.19	-0.11	0.16	2.65	-0.09	0.43	-0.2	0.78	-0.45	-0.68	0.08	-0.88	-2.51
HLG70_02200	*modA*	molybdate ABC transporter substrate-binding protein	0.2	-4.84	-4.85	-6.01	-0.45	-2.91	-3.25	-3.2	-2.8	-3.4	-0.87	-1.27	-0.17
HLG70_02205	*modB*	molybdate ABC transporter permease subunit	0.46	-3.58	-3.49	-4.37	-0.36	-2.08	-2.06	-2.13	-1.82	-2.59	-0.31	-0.45	0.23
HLG70_02210		ATP-binding cassette domain-containing protein	0.08	-2.83	-2.73	-3.19	-0.37	-1.42	-1.24	-1.3	-1.69	-2.1	-0.29	-0.26	0
**Others**															
HLG70_01165		endopeptidase	-0.42	-0.98	-0.64	0.29	-0.62	-2.24	-1.05	0.28	3.03	2.88	1.76	2.55	2.82
**EPS/ capsular**															
HLG70_06320		capsular biosynthesis protein	0.12	-0.06	-0.05	-0.1	-0.54	-1.75	-1.24	0.7	3.12	2.5	1.43	1.87	3.74
HLG70_06325		hypothetical protein	-0.06	0.21	0.04	0.13	-0.4	-1.32	-0.81	0.29	1.96	1.66	0.43	1.04	1.92
HLG70_06330		glycosyltransferase family 2 protein	-0.36	0.28	0.1	0.46	-0.14	-0.69	-0.28	-0.02	1.19	1.46	0.22	0.75	0.52
HLG70_06335		ABC transporter permease	0.05	0.31	0.26	0.2	-0.24	-0.07	0.28	-0.19	1.25	1.01	0.86	1.21	0.67

HLG70_06340	ABC transporter ATP-binding protein	-0.3	0.24	0.04	-0.02	-0.23	-0.72	-0.24	0.19	1.35	1.46	0.38	1	1.37
HLG70_06345	capsule biosynthesis protein	-0.16	0.31	0.17	-0.08	-0.21	-0.64	-0.26	0.25	1.12	1.11	0.16	0.62	1.27
HLG70_06350	glycosyltransferase	-0.06	0.46	0.17	-0.05	-0.22	-0.87	-0.42	0.22	1.52	1.4	0.18	0.86	1.6
HLG70_06355	polysaccharide biosynthesis/export family protein	-0.16	0.37	0.13	-0.05	-0.13	-0.62	-0.31	0.34	1.26	1.34	0.26	0.75	1.46
HLG70_06360	Vi polysaccharide biosynthesis UDP-N-acetylglucosamine C-6 dehydrogenase TviB	0.23	0	-0.09	-0.12	-0.34	-0.82	-0.49	1.03	1.41	0.88	0.59	0.95	2.37
HLG70_06365	capsular polysaccharide biosynthesis protein	-0.17	0.37	0.16	-0.03	-0.16	-0.7	-0.27	0.24	1.23	1.29	0.16	0.75	1.31
HLG70_06370	capsular biosynthesis protein	-0.22	0.34	0.16	-0.04	-0.04	-0.51	-0.12	-0.11	0.96	1.18	0.11	0.61	0.7
HLG70_06375	SDR family NAD(P)-dependent oxidoreductase	-0.18	0.15	-0.05	-0.14	-0.12	-0.55	-0.13	-0.23	0.73	0.84	0.03	0.59	0.45
HLG70_06380	LTA synthase family protein	-0.06	0.33	-0.01	0.07	0.03	-0.41	-0.08	-0.28	0.74	0.88	-0.01	0.61	0.2
HLG70_06385	aminotransferase class I/II-fold pyridoxal phosphate-dependent enzyme	-0.04	0.35	0.12	-0.1	-0.08	-0.5	-0.18	-0.02	1.07	1.08	0.22	0.71	0.97
HLG70_06390	SDR family NAD(P)-dependent oxidoreductase	-0.14	0.33	0.08	-0.06	-0.26	-0.87	-0.47	0.22	1.46	1.39	0.26	0.85	1.55
HLG70_06395	alpha/beta hydrolase	-0.02	0.18	-0.01	0.2	-0.09	-0.35	-0.07	-0.23	0.58	0.55	0.05	0.45	-0.04
**Stress**														
HLG70_14520	CsbD family protein	-0.2	-1.56	-1.16	0.06	-0.56	-2.07	-1.4	-0.13	2.8	2.49	2.29	2.49	2.43
HLG70_14525	DUF1328 domain-containing protein	-0.32	-1.48	-1.24	0.4	-0.49	-1.98	-1.39	-0.23	2.61	2.48	2.1	2.4	1.79
HLG70_14530	BON domain-containing protein	-0.56	-1.75	-1.4	0.29	-0.5	-1.8	-1.24	-0.24	1.14	1.25	1.1	1.23	0.42
**Cell wall**														
HLG70_16010	penicillin acylase family protein	0.03	-0.97	-0.58	-1.77	-0.32	-0.49	-0.83	0.55	1.43	1.13	1.91	1.12	3.56
HLG70_16015	hypothetical protein	-0.38	-1.65	-0.78	-2.1	-0.27	-0.44	-0.79	0.72	0.92	1.08	2.12	0.85	3.54
**Energy**														

**production**														
HLG70_19840	F0F1 ATP synthase subunit epsilon	0.67	-0.32	0.36	-1.18	-0.18	0.55	-0.28	1.65	-0.96	-1.77	-0.1	-1.68	1.68
HLG70_19845	F0F1 ATP synthase subunit beta	0.72	-0.35	0.3	-1.99	-0.22	0.73	-0.53	1.94	-1.1	-2	-0.02	-1.99	2.64
HLG70_19850	F0F1 ATP synthase subunit gamma	0.4	-0.69	0.06	-2.14	-0.29	0.75	-0.57	1.88	-1.54	-2.18	-0.1	-2.23	2.29
HLG70_19855	F0F1 ATP synthase subunit alpha	0.41	-0.55	0.13	-2.27	-0.18	0.69	-0.51	1.86	-1.44	-1.99	-0.2	-2.14	2.5
HLG70_19860	F0F1 ATP synthase subunit delta	0.34	-0.22	0.15	-1.36	-0.07	0.61	-0.3	1.7	-0.98	-1.34	-0.15	-1.5	1.89
HLG70_19865	F0F1 ATP synthase subunit B	0.52	0.04	0.49	-1.43	-0.19	0.79	-0.49	2.22	-0.71	-1.38	0.03	-1.76	2.75
HLG70_19870	F0F1 ATP synthase subunit C	-0.33	-1.14	-0.36	-1.95	-0.14	0.9	-0.29	2.33	-2.66	-2.42	-0.62	-2.65	1.43
HLG70_19875	F0F1 ATP synthase subunit A	-0.36	-0.82	0.23	-0.58	-0.11	0.68	0.2	1.91	-1.19	-0.9	0.31	-1.28	1.11
HLG70_19880	ATP synthase subunit I	-0.15	-0.72	0.34	-0.41	-0.11	-0.09	-0.18	0.82	-0.3	-0.21	0.32	-0.87	0.74
HLG70_19885	DUF1254 domain-containing protein	-0.05	0.12	-0.01	0.02	-0.52	-1.6	-1.06	0.03	3.19	2.76	1.46	2.07	3.01
**Mobilty**														
HLG70_10070	flagellar protein FlaG	-0.39	-1.88	-0.64	-0.48	0.01	0.21	-0.25	0.52	-3.8	-3.35	-1.71	-3.48	-2.99
HLG70_10075	flagellar filament capping protein FliD	0.11	-1.19	0.04	-0.72	0.04	0.71	-0.06	1.19	-3.87	-3.89	-1.97	-4.03	-2.14
HLG70_10080	flagellar export chaperone FliS	-0.34	-1.53	-0.34	-0.19	-0.02	0.63	0.19	0.94	-3.9	-3.54	-1.74	-3.44	-2.96
HLG70_10085	flagellar protein FliT	-0.43	-1.3	-0.38	0.16	0.04	0.3	0.12	0.29	-2.65	-2.14	-1.06	-2.22	-2.71
HLG70_10090	flagellar hook-length control protein FliK	0.13	-0.55	0.2	0.65	-0.08	-0.19	-0.08	0.06	-0.91	-1.07	-0.55	-1.24	-1.69
HLG70_10095	EscU/YscU/HrcU family type III secretion system export apparatus switch protein	-0.39	-0.82	-0.27	0.72	0.04	0.04	0.17	-0.11	-0.94	-0.46	-0.08	-0.56	-1.95
HLG70_10100	flagellar brake protein	-0.12	-0.59	-0.13	-0.1	0.02	0.28	0.05	0.3	-1.41	-1.23	-0.54	-1.3	-1.2
HLG70_10105	flagellar hook-basal body complex protein FliE	-0.25	-1.57	-1.03	-0.41	-0.04	0.68	0.08	0.53	-4.39	-4.13	-2.14	-3.35	-3.63
HLG70_10110	flagellar M-ring protein FliF	0.07	-0.56	0.08	-0.93	0.02	0	0.02	0.13	-1.62	-1.62	-1.06	-1.74	-0.74
HLG70_10115	flagellar motor switch protein FliG	0.12	-0.8	-0.26	-1.84	-0.02	0.57	0.02	0.45	-3	-3.1	-1.63	-2.79	-0.89
HLG70_10120	flagellar assembly protein FliH	0.29	-0.61	-0.19	-1.29	0.01	0.3	-0.13	0.17	-1.95	-2.18	-1.04	-1.96	-0.68

HLG70_10125	flagellar protein export ATPase FliI	0.14	-0.78	-0.39	-0.88	0.05	-0.15	-0.03	-0.13	-1.43	-1.48	-0.81	-1.14	-0.87
HLG70_10130	flagella biosynthesis chaperone FliJ	-0.25	-1.16	-0.79	-1.98	0.02	0.11	0.13	-0.11	-2.32	-2	-1.05	-1.46	-0.65
HLG70_10135	flagellar hook-length control protein FliK	0.42	-0.65	-0.24	-1.66	0.04	0.19	-0.07	0.39	-2.42	-2.76	-1.59	-2.32	-0.56
HLG70_10140	flagellar basal body-associated protein FliL	0.21	-0.63	-0.29	-0.82	-0.07	0.68	0.07	0.76	-3.13	-3.37	-1.82	-2.83	-1.74
HLG70_10145	flagellar motor switch protein FliM	-0.21	-1.17	-0.54	-0.51	-0.01	0.61	0.28	0.58	-3.7	-3.46	-1.92	-2.94	-2.8
HLG70_10150	flagellar motor switch protein FliN	0.37	-0.76	-0.09	-0.57	0.12	0.64	0	0.94	-3.13	-3.33	-1.72	-3.11	-1.81
HLG70_10155	flagellar biosynthetic protein FliO	0.15	-0.44	0.04	-0.18	0.11	-0.08	0.02	0.16	-1.68	-1.68	-1.32	-1.77	-1.53
HLG70_10160	flagellar type III secretion system pore proteinFliP	0	-0.35	0.02	0.49	0.09	-0.06	0.23	-0.06	-0.77	-0.63	-0.49	-0.62	-1.51
HLG70_10165	flagellar biosynthesis protein FliQ	0.04	-0.94	-0.22	0.29	0.01	0.5	0.59	0.4	-2.38	-2.36	-0.94	-1.64	-2.47
HLG70_10170	flagellar biosynthetic protein FliR	0.24	0.18	0.07	0.42	0.04	0.07	0.32	-0.12	0.33	0.17	0.22	0.52	-0.4
HLG70_10175	PAS domain S-box protein	-0.1	-0.3	0.41	-0.62	0.04	-0.13	0.01	-0.07	-0.33	-0.15	-0.17	-0.8	0.03
HLG70_10180	Tar ligand binding domain-containing protein	0.24	-1.06	-0.37	-2.26	0.03	0.71	0	1.05	-3.24	-3.41	-1.47	-2.94	-0.12
HLG70_10185	hypothetical protein	-0.37	-1.37	-0.75	-1.31	0.04	0.6	0.19	0.96	-3.61	-3.15	-1.65	-2.73	-1.53
HLG70_10190	Tar ligand binding domain-containing protein	0.13	-0.64	0.1	-0.79	0.04	0	-0.01	0.36	-1.27	-1.32	-0.63	-1.44	-0.32
HLG70_10195	Tar ligand binding domain-containing protein	0.08	-0.8	-0.06	-0.54	0.02	0.24	0.05	0.64	-1.88	-1.9	-0.84	-1.84	-0.9
HLG70_10200	flagellar hook-associated protein FlgL	0.1	-1.28	-0.25	-0.52	0.05	0.97	0.06	1.36	-4.39	-4.39	-2.14	-4.14	-2.7
HLG70_10205	flagellar hook-associated protein FlgK	-0.01	-1.24	-0.26	-0.38	0	0.91	0.06	1.4	-4.11	-4.06	-1.95	-3.84	-2.52
HLG70_10210	flagellar assembly peptidoglycan hydrolase FlgJ	-0.1	-1.12	-0.44	-0.66	-0.02	0.24	0.02	0.24	-2.83	-2.7	-1.48	-2.44	-2.13
HLG70_10215	flagellar basal body P-ring protein FlgI	0.11	-0.92	-0.25	-0.42	-0.03	0.27	0.01	0.36	-2.5	-2.6	-1.31	-2.3	-1.91
HLG70_10220	flagellar basal body L-ring protein FlgH	0.33	-0.66	-0.14	-0.48	0	0.44	0.08	0.43	-2.7	-2.98	-1.6	-2.54	-1.98

HLG70_10225	flagellar basal-body rod protein FlgG	0.3	-0.93	-0.36	-1.35	0.03	1.09	-0.09	1.1	-4.23	-4.46	-2.21	-4.03	-1.97
HLG70_10230	flagellar basal body rod protein FlgF	0.24	-1.04	-0.44	-1.36	-0.07	0.94	-0.14	0.92	-4.16	-4.43	-2.18	-3.93	-2.07
HLG70_10235	flagellar hook-basal body complex protein	0.28	-0.99	-0.36	-1.58	0.01	1.42	-0.05	1.34	-5.07	-5.3	-2.66	-4.83	-2.34
HLG70_10240	flagellar hook assembly protein FlgD	0.38	-0.74	-0.17	-1.27	0.07	1.65	0.15	1.52	-4.65	-4.92	-2.26	-4.4	-2.05
HLG70_10245	flagellar basal body rod protein FlgC	0.23	-0.84	-0.3	-0.86	-0.02	1.13	0.12	0.99	-4.42	-4.62	-2.45	-4.06	-2.77
HLG70_10250	flagellar basal body rod protein FlgB	-0.1	-1.06	-0.55	-0.44	0.04	0.69	0.04	0.49	-3.91	-3.73	-2.16	-3.39	-3.16
HLG70_10255	flagellar basal body P-ring formation protein FlgA	-0.11	-0.83	-0.45	0.26	-0.03	-0.07	-0.08	0.03	-1.84	-1.71	-1.08	-1.53	-2.26
HLG70_10260	flagellar biosynthesis anti-sigma factor FlgM	-0.48	-1.56	-0.81	0.44	-0.09	-0.14	-0.2	0.3	-2.73	-2.29	-1.32	-2.19	-3.05
HLG70_10265	flagellar protein FlgN	0.24	-0.86	-0.04	0.47	-0.03	-0.02	-0.19	0.54	-1.88	-2.1	-1.05	-2.09	-2
HLG70_10270	flagellar biosynthesis protein FlhF	0.2	-0.71	-0.33	-0.14	0.01	-0.02	-0.04	0.07	-1.61	-1.76	-0.92	-1.38	-1.59
HLG70_10275	flagellar biosynthesis protein FlhA	-0.01	-0.7	-0.32	-0.52	0.01	0.02	0.03	-0.01	-1.55	-1.48	-0.83	-1.26	-1.23
HLG70_10280	flagellar type III secretion system protein FlhB	0.34	-0.55	-0.08	-0.28	-0.01	0.17	0.01	0.05	-1.52	-1.83	-0.8	-1.49	-1.38
HLG70_10285	chemotaxis protein	0.13	0.12	-0.43	-0.95	-0.05	0.69	-0.12	0.25	-0.91	-1.04	-0.34	-0.67	0.1
HLG70_10290	protein phosphatase CheZ	0.5	-0.47	-0.01	-0.92	-0.01	0.8	0.02	1.16	-2.62	-3.09	-1.36	-2.66	-0.73
HLG70_10295	chemotaxis response regulator CheY	0.44	-0.58	-0.04	-0.98	-0.12	1.54	0.12	1.99	-3.74	-4.25	-1.62	-3.65	-0.96
HLG70_10300	chemotaxis response regulator protein-glutamate methylesterase	0.24	-0.61	-0.02	-0.5	-0.01	0.21	0.06	0.59	-1.8	-2.01	-0.98	-1.79	-0.9
HLG70_10305	chemotaxis protein CheR	-0.02	-0.65	0.09	-0.29	-0.05	0.23	0.11	0.5	-1.98	-1.96	-1.1	-2.02	-1.37
HLG70_10310	Tar ligand binding domain-containing protein	0.33	-0.43	0.23	-0.74	0	0.55	0.03	0.98	-2.15	-2.43	-1.17	-2.41	-0.61
HLG70_10315	chemotaxis protein CheW	0.24	-1.07	-0.22	-0.67	-0.04	1.56	0.19	2	-4.36	-4.59	-1.73	-4.02	-1.88
HLG70_10320	chemotaxis protein CheA	0.11	-1	-0.18	-0.64	-0.07	0.86	0	1.25	-3.49	-3.63	-1.64	-3.38	-1.79

HLG70_10325	response regulator	-0.08	-1.08	0.03	0.06	-0.1	0.95	0.03	1.38	-2.96	-2.93	-0.93	-3.02	-1.83
HLG70_10330	flagellar motor protein MotB	0.12	-0.65	0.36	-0.02	0.03	0.5	0.1	1.09	-2.53	-2.57	-1.39	-2.86	-1.62
HLG70_10335	flagellar motor stator protein MotA	0.28	-0.72	0.26	0.33	0.03	0.6	0.18	1.27	-2.74	-2.94	-1.42	-2.89	-2
HLG70_10340	flagellar transcriptional regulator FlhC	-0.36	-1.31	-0.52	1.37	-0.02	0.53	-0.12	0.64	-3.81	-3.43	-1.97	-3.48	-4.73
HLG70_10345	flagellar transcriptional regulator FlhD	-0.65	-1.6	-0.89	1	-0.07	1.18	-0.36	0.93	-5.18	-4.56	-2.41	-4.71	-5.44
HLG70_10350	RNA polymerase sigma factor FliA	0.04	-0.84	-0.21	0.19	-0.1	0.19	0.03	0.06	-1.85	-1.95	-0.83	-1.68	-2.17
HLG70_10355	flagellin FliC	-0.02	-1.59	-0.41	-0.36	0.11	0.63	-0.14	0.82	-4.6	-4.43	-2.39	-4.39	-3.61
HLG70_10360	class I SAM-dependent methyltransferase	0.1	-1.03	-0.46	-0.64	0.05	1.81	-0.05	2.02	-4.81	-4.82	-1.97	-4.47	-2.33
HLG70_10365	methyltransferase regulatory domain-containing protein	-0.39	-1.4	-0.75	-0.36	-0.03	1.06	0.28	0.94	-4.72	-4.31	-2.27	-3.75	-3.61
HLG70_10370	glucose-1-phosphate cytidylyltransferase	0.2	-0.64	-0.24	0.14	0.03	0.29	0.14	0.36	-2.25	-2.37	-1.31	-1.93	-2.21
HLG70_10375	CDP-glucose 4,6-dehydratase	0.01	-0.47	-0.14	0.25	0	-0.16	-0.05	-0.02	-1.1	-1.06	-0.79	-1.08	-1.56
HLG70_10380	dTDP-4-dehydrorhamnose 3,5-epimerase	-0.05	-0.6	-0.23	0.15	0.06	-0.1	0.02	-0.16	-1.24	-1.08	-0.74	-1.06	-1.74
HLG70_10385	class I SAM-dependent methyltransferase	0.17	-0.55	-0.15	0.11	0.04	0.02	0.08	0.14	-1.73	-1.81	-1.17	-1.56	-1.89
HLG70_10390	NAD-dependent epimerase/dehydratase family protein	-0.15	-0.62	-0.31	0.08	0.1	-0.06	0.06	-0.04	-1.27	-0.98	-0.71	-0.97	-1.58
HLG70_10395	cephalosporin hydroxylase family protein	0.18	-0.79	-0.4	-0.44	-0.06	0.72	0.18	0.83	-3.49	-3.69	-1.97	-2.97	-2.4
HLG70_10400	acyltransferase	0.14	-0.55	-0.25	-0.05	-0.06	0.04	0.02	0.05	-1.71	-1.88	-1.13	-1.51	-1.8
HLG70_10405	phytanoyl-CoA dioxygenase family protein	-0.05	-0.6	-0.34	0.38	-0.03	0.18	0.2	0.13	-1.83	-1.76	-1.06	-1.36	-2.28
HLG70_10410	class I SAM-dependent methyltransferase	-0.31	-0.75	-0.45	0.46	-0.08	0.05	0.11	0.16	-1.6	-1.32	-0.8	-1.11	-2.09
HLG70_10415	hypothetical protein	-0.02	-0.28	-0.19	0.74	0.01	-0.15	-0.07	-0.28	-0.36	-0.29	-0.23	-0.3	-1.57
HLG70_10420	putative motility protein	0.11	-0.95	-0.33	0.69	-0.01	0.28	-0.08	0.78	-2.89	-2.97	-1.65	-2.7	-2.98
**Ribosomal 1**														
HLG70_21890	elongation factor Tu	0.04	-1.45	-0.1	-3.43	-0.32	0.61	-0.4	2.26	-2.04	-2.36	0.01	-2.39	3.47
HLG70_21900	preprotein translocase subunit SecE	-0.61	-1.27	-0.36	-1.31	-0.09	0.56	0.09	1.57	-1.65	-1.08	0.18	-1.26	1.04

HLG70_21905	transcription termination/antitermination protein NusG	-0.38	-1.29	-0.1	-1.62	-0.12	0.93	-0.2	2.39	-1.85	-1.54	0.37	-2.01	1.97
HLG70_21910	50S ribosomal protein L11	-0.13	-1.26	-0.13	-0.56	-0.2	0.77	-0.04	2.07	-1.83	-1.85	0.19	-1.8	0.62
HLG70_21915	50S ribosomal protein L1	0.35	-0.76	0.27	-1.79	-0.27	0.82	-0.24	2.14	-0.89	-1.47	0.69	-1.47	2.84
HLG70_21920	50S ribosomal protein L10	0.34	-0.89	-0.11	0.92	-0.36	1.57	-0.35	3.04	-1.14	-1.79	1.32	-1.45	0.79
HLG70_21925	50S ribosomal protein L7/L12	0.41	-0.76	-0.24	0.68	-0.22	1.42	-0.25	2.83	-1	-1.59	1.19	-1.07	0.96
HLG70_21930	DNA-directed RNA polymerase subunit beta	0.51	-1.05	-0.18	-2.62	-0.26	0.75	-0.25	1.95	-1.39	-2.12	0.41	-1.53	2.99
HLG70_21935	DNA-directed RNA polymerase subunit beta'	0.58	-1.09	-0.1	-2.18	-0.22	0.81	-0.18	2.09	-1.43	-2.18	0.47	-1.57	2.66
**Ribosomal 2**														
HLG70_21970	30S ribosomal protein S12	-0.59	-1.64	-0.35	-1.01	-0.19	1.03	0.42	2.93	-2.9	-2.45	-0.23	-2.2	0.85
HLG70_21975	30S ribosomal protein S7	0.12	-0.68	0.44	-1.5	-0.17	0.97	0.13	2.85	-1.27	-1.52	0.37	-1.64	2.89
HLG70_21980	elongation factor G	0.21	-1.03	0.17	-1.92	-0.27	0.85	-0.15	2.49	-1.62	-2.05	0.25	-2.01	2.59
HLG70_21985	elongation factor Tu	0.32	-1.1	0.03	-2.27	-0.33	0.71	-0.36	2.28	-1.53	-2.13	0.28	-1.98	2.83
HLG70_21990	30S ribosomal protein S10	-0.11	-1.23	-0.61	-1.87	0.03	0.79	0.24	2.01	-2.12	-1.93	-0.1	-1.33	1.57
HLG70_21995	50S ribosomal protein L3	-0.25	-1.21	0.37	-1.43	-0.17	0.73	0.33	2.71	-1.44	-1.32	0.5	-1.54	2.5
HLG70_22000	50S ribosomal protein L4	0.01	-1.26	0.53	-1.7	-0.24	1.29	0.41	3.33	-1.22	-1.43	1.32	-1.4	3.62
HLG70_22005	50S ribosomal protein L23	-0.37	-1.82	0.18	-1.71	-0.18	1.36	-0.02	3.54	-2.41	-2.17	0.76	-2.68	2.66
HLG70_22010	50S ribosomal protein L2	-0.03	-1.15	0.19	-1.02	-0.08	1.21	0.27	2.73	-1.31	-1.31	1.05	-1.29	2.26
HLG70_22015	30S ribosomal protein S19	-0.22	-1.88	-0.31	-1.04	-0.11	1.67	0	3.08	-2.61	-2.45	0.93	-2.37	1.32
HLG70_22020	50S ribosomal protein L22	0.46	-1.38	0.23	-0.68	-0.16	1.85	0.18	3.45	-1.86	-2.44	1.36	-1.98	2.07
HLG70_22025	30S ribosomal protein S3	0.43	-1.12	0.1	-0.67	-0.09	1.5	0.07	2.81	-1.28	-1.76	1.33	-1.38	2.01
HLG70_22030	50S ribosomal protein L16	0.22	-1.34	-0.14	-0.75	-0.17	1.26	0.12	2.53	-1.74	-2.08	0.86	-1.55	1.35
HLG70_22035	50S ribosomal protein L29	0.02	-2.05	-0.52	-0.52	0.17	1.91	0.19	3.39	-3.11	-2.91	0.84	-2.46	0.61

HLG70_22040	30S ribosomal protein S17	-0.25	-2.08	-0.9	-0.14	0.22	1.71	0.3	3.08	-3.25	-2.73	0.54	-2.11	-0.22
**Ribosomal 3**														
HLG70_22105	50S ribosomal protein L14	0.03	-1.36	0.32	-1.17	-0.45	0.93	-0.17	3.59	-1.69	-2.12	0.6	-2.24	2.88
HLG70_22110	50S ribosomal protein L24	0.31	-0.94	0.93	-1.74	-0.29	1.16	0.09	3.77	-1.44	-2	0.66	-2.36	3.88
HLG70_22115	50S ribosomal protein L5	-0.04	-1.06	0.61	-2.08	-0.18	1.16	-0.14	3.49	-2.09	-2.18	0.13	-2.91	3.28
HLG70_22120	30S ribosomal protein S14	-0.29	-1.35	0.29	-2.25	-0.28	1.13	-0.13	3.27	-2.4	-2.34	0.08	-2.88	2.93
HLG70_22125	30S ribosomal protein S8	-0.35	-1.31	0.09	-2.38	-0.31	0.95	-0.01	2.93	-2.36	-2.28	-0.1	-2.53	2.77
HLG70_22130	50S ribosomal protein L6	-0.03	-1.09	0.09	-2.26	-0.19	0.92	-0.06	2.65	-1.92	-2.04	0.09	-2.14	2.8
HLG70_22135	50S ribosomal protein L18	0.08	-1.08	-0.19	-1.81	-0.22	0.7	-0.35	2.19	-1.61	-1.86	0.17	-1.84	2.2
HLG70_22140	30S ribosomal protein S5	0.36	-1.01	-0.21	-1.34	-0.34	0.69	-0.62	2.13	-1.13	-1.79	0.57	-1.61	2.15
HLG70_22145	50S ribosomal protein L30	0.06	-1.45	-0.6	-1.46	-0.14	0.86	-0.21	2.18	-2.09	-2.25	0.22	-1.76	1.35
HLG70_22150	50S ribosomal protein L15	0.33	-1.13	-0.28	-0.88	-0.24	0.72	-0.3	1.94	-1.21	-1.75	0.63	-1.3	1.42
HLG70_22155	preprotein translocase subunit SecY	0.32	-1.15	-0.27	-0.06	-0.36	0.8	-0.6	2.28	-1.26	-1.9	0.68	-1.66	0.9
HLG70_22160	translation initiation factor IF-1	-0.99	-2.28	-1.41	-1.14	-0.11	0.46	-0.31	1.84	-3.31	-2.39	-0.58	-2.27	-0.52
HLG70_22165	50S ribosomal protein L36	-1.39	-2.65	-1.79	-0.79	0.01	0.69	-0.07	1.68	-4.2	-2.76	-0.86	-2.54	-1.92
HLG70_22170	30S ribosomal protein S13	0.39	-0.83	-0.09	-0.61	-0.3	0.64	-0.54	1.94	-1.4	-2.04	0.07	-1.92	0.97
HLG70_22175	30S ribosomal protein S11	0.13	-1	-0.33	-0.94	-0.22	0.51	-0.3	1.59	-1.73	-2.04	-0.22	-1.77	0.6
HLG70_22180	30S ribosomal protein S4	0.52	-0.84	0.03	-1.35	-0.27	0.76	-0.44	1.97	-1.67	-2.42	-0.07	-2.2	1.46
HLG70_22185	DNA-directed RNA polymerase subunit alpha	0.29	-0.75	0.05	-2	-0.13	0.74	-0.14	2.05	-1.07	-1.45	0.41	-1.33	2.8
HLG70_22190	50S ribosomal protein L17	0.23	-1.15	-0.24	-1.33	-0.17	0.48	-0.13	1.58	-1.65	-2	-0.02	-1.61	1.08

Note：1, WAs0_2vsWCK; 2, WAs2vsWCK; 3 , WSb0_05vsWCK; 4, WSb0_5vsWCK; 5, SAs0_2vsSCK; 6, SAs2vsSCK; 7, SSb0_05vsSCK; 8, SSb0_5vsSCK; 9, SCKvsWCK; 10, SAs0_2vsWAs0_2; 11, SAs2vsWAs2; 12, SSb0_05vsWSb0_05; 13, SSb0_5vsWSb0_5.
